# Tumor microenvironment modulation enhances immunologic benefit of chemoradiotherapy

**DOI:** 10.1186/s40425-018-0485-9

**Published:** 2019-01-15

**Authors:** Aurelie Hanoteau, Jared M. Newton, Rosemarie Krupar, Chen Huang, Hsuan-Chen Liu, Angelina Gaspero, Robyn D. Gartrell, Yvonne M. Saenger, Thomas D. Hart, Saskia J. Santegoets, Damya Laoui, Chad Spanos, Falguni Parikh, Padmini Jayaraman, Bing Zhang, Sjoerd H. Van der Burg, Jo A. Van Ginderachter, Cornelis J. M. Melief, Andrew G. Sikora

**Affiliations:** 1 Department of Otolaryngology-Head and Neck surgery, Baylor College of Medicine, Houston, TX USA; 2 Interdepartmental Program in Translational Biology and Molecular Medicine, Baylor College of Medicine, Houston, TX USA; 3 Pathology of the University Hospital Schleswig-Holstein, Campus Luebeck and Research Center Borstel, Leibniz Lung Center, Lubeck and Borstel, Germany; 4 Department of Molecular and Human Genetics, Baylor College of Medicine, Houston, TX USA; 5 Lester & Sue Smith Breast Center, Baylor College of Medicine, Houston, TX USA; 6 Department of Pediatrics, Division of Pediatric Hematology/Oncology, Columbia University Irving Medical Center/New York Presbyterian, New York, USA; 7 Department of Medicine, Division of Hematology/Oncology, Columbia University Irving Medical Center/New York Presbyterian, New York, USA; 8 Department of Medical Oncology, Leiden University Medical Center, Leiden, The Netherlands; 9 Laboratory of Cellular and Molecular Immunology, Vrije Universiteit Brussel (VUB), Brussels, Belgium; 10 Laboratory of Myeloid Cell Immunology, VIB Center for Inflammation Research, Brussels, Belgium; 11 Department of Surgery, University of South Dakota Sanford School of Medicine, Vermillion, SD USA; 12 ISA Pharmaceuticals, Leiden, The Netherlands; 13 Department of Cell and Gene Therapy, Baylor College of Medicine, Houston, TX USA

**Keywords:** Immunotherapy, Tumor microenvironment, Inducible nitric oxide synthase (iNOS), Cyclophosphamide, L-n6-(1-iminoethyl)-lysine (L-NIL), Chemoradiotherapy, Radiotherapy, Head and neck squamous cell carcinoma, Head and neck cancer, Human papillomavirus (HPV)

## Abstract

**Background:**

Chemoradiotherapy (CRT) remains one of the most common cancer treatment modalities, and recent data suggest that CRT is maximally effective when there is generation of an anti-tumoral immune response. However, CRT has also been shown to promote immunosuppressive mechanisms which must be blocked or reversed to maximize its immune stimulating effects.

**Methods:**

Therefore, using a preclinical model of human papillomavirus (HPV)-associated head and neck squamous cell carcinoma (HNSCC), we developed a clinically relevant therapy combining CRT and two existing immunomodulatory drugs: cyclophosphamide (CTX) and the small molecule inducible nitric oxide synthase (iNOS) inhibitor L-n6-(1-iminoethyl)-lysine (L-NIL). In this model, we treated the syngeneic HPV-HNSCC mEER tumor-bearing mice with fractionated (10 fractions of 3 Gy) tumor-directed radiation and weekly cisplatin administration. We compared the immune responses induced by CRT and those induced by combinatory treatment (CRT + CTX/L-NIL) with flow cytometry, quantitative multiplex immunofluorescence and by profiling immune-related gene expression changes.

**Results:**

We show that combination treatment favorably remodels the tumor myeloid immune microenvironment including an increase in anti-tumor immune cell types (inflammatory monocytes and M1-like macrophages) and a decrease in immunosuppressive granulocytic myeloid-derived suppressor cells (MDSCs). Intratumoral T cell infiltration and tumor antigen specificity of T cells were also improved, including a 31.8-fold increase in the CD8^+^ T cell/ regulatory T cell ratio and a significant increase in tumor antigen-specific CD8^+^ T cells compared to CRT alone. CTX/LNIL immunomodulation was also shown to significantly improve CRT efficacy, leading to rejection of 21% established tumors in a CD8-dependent manner.

**Conclusions:**

Overall, these data show that modulation of the tumor immune microenvironment with CTX/L-NIL enhances susceptibility of treatment-refractory tumors to CRT. The combination of tumor immune microenvironment modulation with CRT constitutes a translationally relevant approach to enhance CRT efficacy through enhanced immune activation.

**Electronic supplementary material:**

The online version of this article (10.1186/s40425-018-0485-9) contains supplementary material, which is available to authorized users.

## Background

Head and neck squamous cell carcinoma (HNSCC) is the 6th most common cancer worldwide and has a poor prognosis at advanced stages of disease [1]. Human papillomavirus (HPV)-associated cancer of the oropharynx (throat) has become the fastest-increasing HNSCC subtype in the US and other developed countries, with the HPV16 viral type accounting for roughly 80% of HPV-positive HNSCC (HPV-HNSCC) [2]. Transformation of epithelial cancer cells by HPV16 depends on expression of the onco-viral proteins, E6 and E7 [3], which have also been shown to enhance intratumoral CD8^+^ T cell infiltration [4]. This enhanced immune response likely contributes to improved response and survival rates of HPV-HNSCC after chemoradiotherapy (CRT) compared to HPV-negative HNSCC [5] highlighting the potential role of tumor immune microenvironment as a determinant of CRT treatment response.

The standard-of-care CRT, consisting of tumor-directed radiotherapy and concurrent platinum-based chemotherapy (cisplatin or carboplatin) [6], is highly effective for the majority of primary HPV-HNSCC patients, but exhibits high failure rates in patients with locoregionally advanced, and recurrent or metastatic disease [7, 8]. Increasing evidence suggests that CRT simultaneously induces pro- and anti-tumoral immune responses [9]. CRT reportedly favors a number of anti-tumor mechanisms such as (i) improved antigen cross-presentation, (ii) increased Type I interferon release, and (iii) enhanced *major histocompatibility complex* (MHC) class I expression on tumor cells [10, 11]. However, it has also been linked to a variety of immunosuppressive effects including (i) the development of chemotherapy-resistant regulatory T cells (Tregs) [12], (ii) increased levels of circulating MDSCs (iii) the depletion and exhaustion of tumor-reactive T cells [13], and (iv) inhibition of T cell reactivity [14]. The multi-faceted immunomodulatory effects induced by CRT are limiting factors in its ability to stimulate effective immunological responses against solid tumors. Thus, immunomodulation of the tumor microenvironment is a promising approach to enhance the efficacy of CRT in solid tumors.

During cancer development, the tumor-mediated aberrant expression of inflammatory molecules contributes to the induction and intratumoral infiltration of immunosuppressive cells, such as MDSCs and Tregs. One such inflammatory mediator, inducible nitric oxide synthase (iNOS), is highly upregulated in numerous solid tumors [15, 16], and favors tumor growth through the enhanced induction and recruitment of MDSCs [17]. iNOS inhibition, such as with the iNOS-selective small molecule inhibitor L-n6-(1-iminoethyl)-lysine (L-NIL) [18] which has previously been tested in clinical trials for asthma and inflammatory disease [19], induces both immune-dependent and independent anti-tumor effects. However, we have demonstrated that iNOS inhibition also increases Treg development and suppressive function [20]. To address this, we determined that cyclophosphamide (CTX) is an ideal complement to iNOS inhibition due to its ability to deplete Tregs [21]. Additionally, CTX enhances T cell activity and specificity by changing the T cell receptor (TcR) clonality [22–24]. We further demonstrated that the therapeutic combination of CTX with L-NIL decreases intratumoral MDSC and Treg levels and increases CD8^+^ T cell infiltration [20], leading to enhanced anti-tumor effects. This suggests that the combination of CTX/LNIL can positively re-condition the tumor immune microenvironment, with the potential to enhance the efficacy of other therapeutic regimens, such as CRT. We therefore hypothesized that adjuvant CTX/L-NIL could reverse the hostile tumor microenvironment, thereby enhancing the immunologic benefit of CRT.

To test this hypothesis, we used a syngeneic murine tumor model of HPV-HNSCC (mEER) featuring murine pharyngeal epithelial cells transformed with HPV16 E6 and E7 oncogenes and H*-*ras [25]. The mEER tumor model was chosen because its response to CRT has previously been shown to depend on an intact immune response [26] and it contains viral antigens (HPV16 E6/E7) suitable for monitoring antigen-specific T cell responses. Herein, we show that, while CTX/L-NIL or CRT alone induce modest tumor regression their combination significantly improved treatment efficacy, leading to an average of 21% complete tumor rejection in a CD8^+^ T cell-dependent manner. This enhanced response was attributed to significant improvement in the tumor immune microenvironment including (i) favorable alterations in the tumor myeloid compartment, (ii) an increase in the ratio of CD8^+^ T cells/ Tregs and (iii) increased infiltration of HPV16 E7-specific CD8^+^ T cells. These results suggest that modulation of the tumor immune microenvironment is an effective approach to improve treatment efficacy of conventional CRT in HPV-HNSSC and other solid tumors.

## Methods

### Mice

C57BL/6 J male mice were purchased from The Jackson Laboratory and housed under specific pathogen-free conditions in standard temperature and lighting conditions with free access to food and water. Tumor inoculation was performed when mice reached 8–10 weeks of age. All experiments were performed with approval of the Institutional Animal Care and Use Committee (IACUC) at Baylor College of Medicine (BCM) and followed established protocols.

### Tumor models

MEER tumor cell line expressing HPV16 E6, E7 and hRas was obtained from Dr. Chad Spanos at the Sanford Research center/ University of South Dakota and maintained in E-media as previously described [26]. C57BL/6 J mice were injected subcutaneously (s.c.) with 10^6^ mEER cells in the flank.

MOC2 cell line was a generous gift from Dr. Ravindera Uppaluri at Brigham and Women’s Hospital/ Harvard Medical School and maintained as previously described [27]. C57BL/6 J mice were injected subcutaneously (s.c.) with 15X10^4^ MOC2 cells in the flank.

Mice were monitored twice a week for tumor growth. Measurements were performed by using calipers and tumor area (mm^2^) is expressed as L x W, where L is Length and W is Width. Growth curve experiments were stopped once tumors reached 225 mm^2^.

### In vivo treatment

All mice were randomized prior to treatment. Once tumors become established (average tumor size of 65 mm^2^ or 19 mm^2^, day 17–18 or 8 after mEER and MOC2 inoculation, respectively) treatment was initiated over two weeks. The chemoradiotherapy treatment combined a fractionated tumor-directed radiation regimen (10 X 3Gy daily) (see Supplementary Material for further details) with weekly cisplatin (83μg/mouse; Selleck Chemicals) intraperitoneal injections. The immunomodulatory treatment combined weekly intraperitoneal injections of cyclophosphamide (2 mg/mouse; TCI Chemicals) and continuous oral administration of L-NIL (0.2%; Enzo Life Sciences) in the drinking water ad libitum.

For CD8 depletion experiments, all mice received the combinatory treatment and were injected with 1 mg depleting *InVivo*MAb anti-mouse CD8α (clone 53–6.7; BioXCell) or *InVivo*MAb rat IgG2a isotype control (clone 2A3; BioXCell) 2 days prior the treatment, and further treated with weekly 250 μg antibodies for 3 consecutive weeks.

### Human primary tumor Immunophenotyping

The 9 patients included in this study were part of a larger (*N* = 51) published cohort of HPV^+^ OPSCC patients [28, 29]. All 9 patients included in this study received standard-of-care adjuvant (chemo) radiotherapy following surgical resection of their primary tumor. An overview of patient characteristics and treatments received is available in the prior publication [29]. Patients were involved after signing informed consent and studies were conducted in accordance with the Declaration of Helsinki and approved by the local medical ethical committee of the Leiden University Medical Center (LUMC) and in agreement with the Dutch law.

Following tumor resection, high-dimensional single cell mass cytometry (cyTOF) analysis and functional studies were performed to analyze immune cell population tumor infiltration and cytokine of the general tumor infiltrating lymphocyte. The tumor microenvironment was reanalyzed for the relative low and strong presence of immune cell phenotypes and grouped according to the presence (immune response positive (IR+)) or absence (immune response negative- (IR-)) of HPV16-specific T cell tumor infiltration [28, 29].

### Flow cytometry

To characterize tumor immune cell infiltration, mEER tumors were harvested, digested and stained using the method previously described [30]. Briefly, tumors were digested in RPMI 1640 (Sigma-Aldrich) containing DNase I (20 U/ml; Sigma-Aldrich), Collagenase I (1 mg/ml; EMD Millipore) and Collagenase IV (250 U/ml; Worthington Biochemical Corporation) prior to mechanical disaggregation to form single cell suspensions. Following digestion, tumor infiltrating leukocytes were enriched using Lymphoprep™ (STEMCELL Technologies). Single cell suspensions were also prepared from tumor-draining inguinal lymph node and spleen with additional lysis of splenic red blood cells (RBC) using RBC lysis buffer (Invitrogen). Leukocytes were blocked with anti-mouse CD16/CD32 Fc block (BD Biosciences) and stained using one of various antibody panels (Additional file 1: Table S1 and S2). The viability of cells was determined using LIVE/DEAD™ Fixable Blue Dead Cell Stain Kit (Invitrogen). For intracellular staining, cells were fixed and permeabilized with Intracellular Fixation and Permeabilization Buffer Set (eBioscience) and stained using the appropriate antibody panel (Additional file 1: Table S1). Data were acquired on a LSRII and LSR Fortessa (BD Biosciences) flow cytometers, for myeloid and T cell panels respectively, and analyzed using FlowJo v10 software (FlowJo, LLC).

### Quantitative multiplex immunofluorescence

Sectioning and Staining: After harvesting, tumors were immediately fixed overnight in 10% neutral-buffered formalin. Fixed tumors were embedded in paraffin and sections were cut at a thickness of 5 μm. Full-section slides of tumor tissues were stained using Opal multiplex 6-plex kits, according to the manufacturer’s protocol (PerkinElmer), for DAPI, Epcam (polyclonal; Abcam, 1:100 dilution), CD3 (clone SP7; Spring Biosciences; 1:100 dilution), CD8 (clone 4SM15; ThermoFisher; 1:500), CD4 (clone 4SM95; eBioscience, 1:50), Foxp3 (polyclonal; ThermoFisher, 1:500), and Granzyme B (polyclonal; Abcam, 1:200). Single color controls and an unstained slide were also included.

Multispectral imaging and analysis: Multispectral image capture was done at 20X magnification using Vectra (PerkinElmer, Hopkinton, MA). Images were analyzed using inForm software version 2.4.1 (PerkinElmer) as previously described [31]. Further details are presented in the Supplementary Materials.

### Gene expression analysis

Tumor samples were harvested and flash frozen in liquid nitrogen. Total RNA was extracted with the RNeasy Mini Kit (Qiagen) as per the manufacturer’s instructions. Gene expression profiling was performed on 100 ng RNA using the nCounter® PanCancer Immune Profiling Panel (NanoString Technologies, Inc) containing 770 genes involved in cancer immune response. Further details are presented in the Additional file 1.

### In vitro CD8^+^ T cell cytotoxicity assay

To observe whether changes in the tumor myeloid compartment after treatment influence intratumoral CD8^+^ T cell cytotoxicity, CD8^+^ T cells were purified from the spleen of naïve C57BL/6 J mice with a magnetic bead-based CD8^+^ T cell negative selection kit (Miltenyi Biotec) and CD11b^+^/CD11c^+^ cells were isolated from mEER tumors undergoing treatment using a magnetic bead-based CD11c and CD11b positive selection kit (Miltenyi Biotec). 10^5^ CD8^+^ T cells were co-cultured with 3X10^4^ CD11b^+^/CD11c^+^ cells in enriched DMEM (20% FBS, 2 mM L-glutamine, 1% non-essential amino acid, 1 mM sodium pyruvate, 50 nM 2-mercaptoethanol, 1% penicillin/ streptomycin; as previously described [32]) including 10 ng/ml IL-2 (BioLegend), 2 μg/ml anti-CD3 (clone: 145-2C11; BioLegend) and 5 μg/ml anti-CD28 (clone: 37.51; BioLegend). After 4 days of co-culture, CD8^+^ T cells were collected and co-cultured with 3X10^3^ mEER tumor cells at a ratio of 4:1 (CD8^+^ T cell: tumor) in enriched DMEM including IL-2 (10 ng/ml). After 24 h, tumor cell apoptosis was observed via Cytation Cell Imaging Reader (Biotek) and quantified via flow cytometry using an Annexin V/Dead Cell Apoptosis kit (Invitrogen).

### Statistical analysis

Data sets were tested for Gaussian distribution using the D’Agostino-Pearson normality test. For parametric data sets, statistical significance was determined by: unpaired t test for two-tailed data and ANOVA test followed by selected comparison by Tukey’s multiple comparison tests with multiple comparison correction. For non-parametric data sets, statistical significance was determined by: Mann-Whitney test for two tailed data and Kruskal-Wallis test followed by selected comparison by Dunn’s multiple comparison tests with multiple comparison correction. Survival was analyzed by the Kaplan– Meier method using Log-rank test. (*p < 0.05; **p < 0.01; ***p < 0.001; ****p < 0.0001; ns, non-significant). Outliers from flow cytometry analysis were determined using ROUT (Q = 1%) method.

## Results

### Tumor immune microenvironment remains “cold” following CRT

**Fig. 1:**
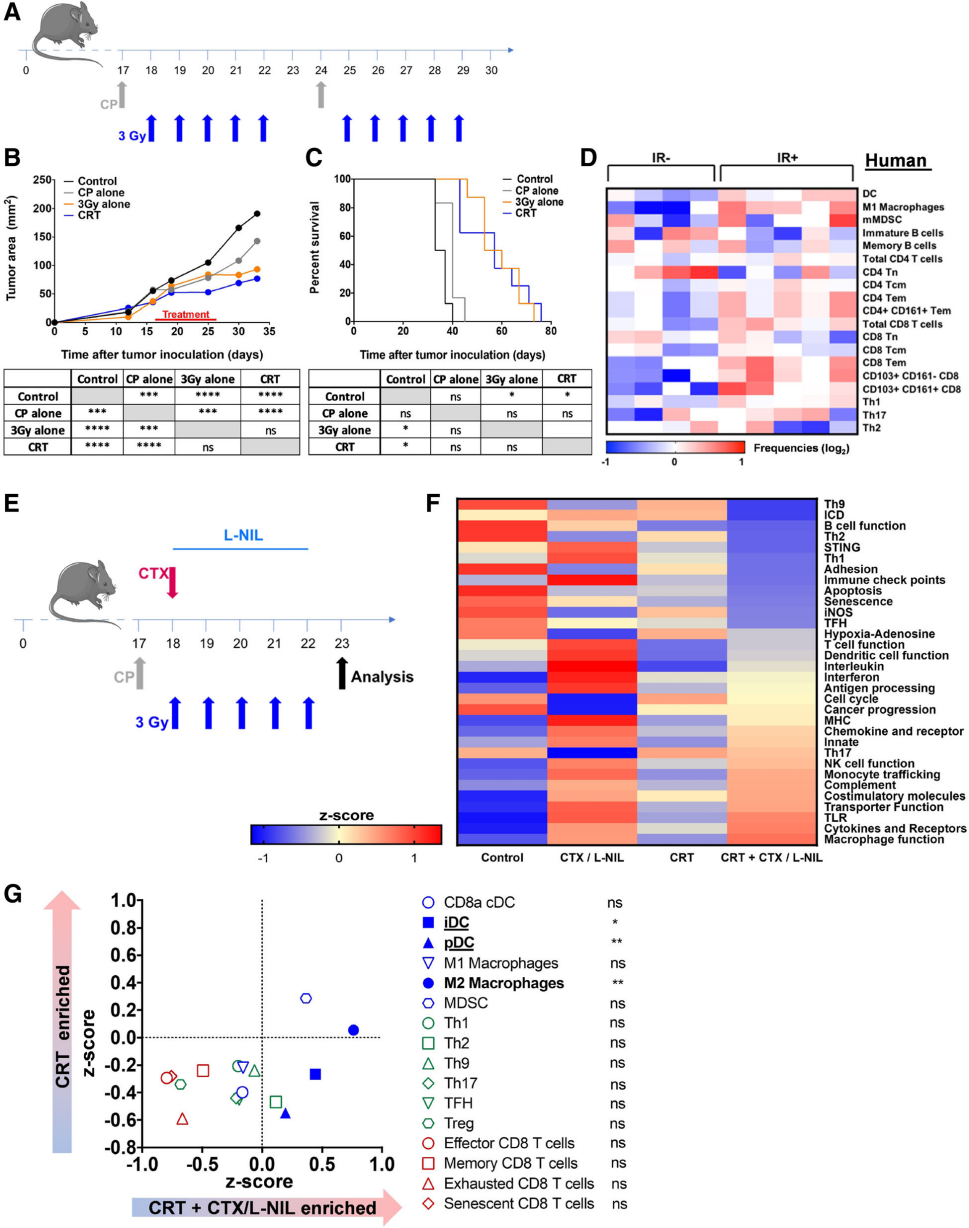
CTX / L-NIL reverses the unfavorable immune microenvironment of CRT treated tumors. **a-c** Subcutaneous established mEER tumors (day 17–18 post tumor cell injection) were treated with tumor-directed radiation (5 X 3Gy) and/or weekly cisplatin (83 μg/mouse) i.p. injections, according to the schedule in (**a**); mice were euthanized when tumors reached 225 mm^2^. **b** Average tumor area (top) and statistical comparison of tumor sizes at time of first euthanasia (bottom; Tukey’s multiple comparison test). **c** Survival curves (top) and statistical comparison between treatments (bottom; Log-rank test); (**b** and **c**; *N* = 1 representative of 2; *n* = 6–8/group). **d** Heatmap depicting the relative abundance of various immune cell populations from CyTOF analysis in HPV16 immune reactive (IR+) and non-reactive (IR-) OPSCC human tumors based on the presence of HPV16-specific tumor infiltrating lymphocytes (TIL). Frequencies of DC (CD14-HLADR^+^CD11c^+^), M1 macrophages (CD163^−^CD14^+^HLA-DR^+^), monocytic MDCS (mMDSC; CD14^+^HLADR^−^), immature B cells (IgM^+^CD38^+^CD20^+^), memory B cells (IgM^−^CD38^−^CD20^+^), total CD4 and CD8^+^ T cells, naïve (Tn; CD45RA^+^CCR7^+^), central memory (Tcm; CD45RA^−^CCR7^+^) and effector memory (Tem; CD45RA^−^CCR7^−^) CD4^+^ and CD8^+^ T cells, CD4^+^CD161^+^ Tem, CD103^+^CD161^−^ and CD103^+^CD161^+^ CD8^+^ T cells. Th-denotation in indicates amount of IFNγ (Th1), IL-5 (Th2) and IL-17A (Th17) produced by the total TIL culture upon phytohemagglutinin stimulation. All data depicted as log-transformed values (base 2) relative to the median for each individual parameter and each column represents an individual patient (*n* = 9 total patients). **e-f** Established mEER tumors were treated with CRT and/or CTX/L-NIL immunomodulation (CTX at 2 mg/mouse i.p. and L-NIL at 0.2% in drinking water) and total tumor RNA was extracted and processed for gene expression analysis after 1 week of treatment, according to schedule in (**e**). **f** Heatmap of differential immune gene-set pathway enrichment represented as z-scores between treatment groups (See Additional file 1: Table S3 for immune pathway gene list). **g** Gene-set based immune cell type enrichment comparing CRT and CRT + CTX/L-NIL represented as z-scores (left; See Additional file 1: Table S4 for immune cell type gene list) and statistical comparison for each immune cell type (right; unpaired t test); (**e** and **f**; *N* = 1; n = 9/group). *p < 0.05; **p < 0.01; ***p < 0.001; ****p < 0.0001; ns, not significant

The immunosuppressive effects of CRT and attempts to overcome them have been well documented [33, 34]; however, development of immunomodulatory strategies capable of reversing the balance of CRT immune effects towards activation remains a critical need. To address this, we developed an immunomodulatory strategy capable of rendering the tumor immune microenvironment susceptible to CRT through a “cold” to a “hot” immunologic transition (Fig. 1, Additional file 1: Figure S1). In this study, immunologically “cold” tumors are best characterized by high immunosuppressive cell infiltrate (i.e. MDSC, Tregs), low anti-tumor immune cell infiltrate (CD8^+^ T cells, M1 macrophages, dendritic cells (DCs)), and a lack of immune-favorable gene expression. Alternatively, immunologically “hot” tumors present with favorable effector/suppressor immune cell ratios and evidence of anti-tumor immune activation.

To better understand the immune impact of CRT on the tumor immune microenvironment, we developed a clinically relevant treatment schedule based on the standard-of-care CRT regimen for HPV-HNSCC. Briefly, fractionated tumor-directed radiation (3 Gy X 10 fractions of radiation) was combined with weekly cisplatin for a total of 2 weeks (Fig. 1a). mEER cells were inoculated subcutaneously in the flank of mice and treatment was initiated once the established tumor reached approximately 65 mm^2^ in size. CRT treatment of tumors induced modest decreases in tumor growth (Fig. 1b) and improvement in survival (Fig. 1c). Tumor gene expression analysis indicates that the tumor microenvironment remains immunologically “cold” at the midpoint of treatment (one week; Fig. 1f). Multiple immunologic gene sets commonly associated with anti-tumor responses (i.e. T cell and dendritic cell (DC) function, interleukins (ILs), antigen processing, MHC, chemokines and receptors) showed low levels of expression similar to those of untreated tumors (Fig. 1f).

### Immune-responsive HPV16^+^ HNSCC human tumors display a “hot” immune microenvironment and improved treatment response

Based on the murine gene expression analysis we next assessed, in a cohort of human HPV16^+^ oropharyngeal squamous cell carcinoma (OPSCC) patients, whether a favorable tumor immune microenvironment could influence standard-of-care treatment benefit (Fig. 1d). Briefly, the 9 patients included in this study received standard-of-care (chemo) radiotherapy following surgical resection of their primary tumor. Patients were grouped based on the presence (immune response positive (IR+)) or absence (immune response negative- (IR-)) of HPV16-specific T cell tumor infiltration observed prior to treatment as previously described [28, 29]. In a prior study, IR+ OPSCC patients treated with surgery followed by adjuvant CRT were shown to have a 3-fold improvement in 5-year survival compared to IR- (ca. 90% vs 30%) [28, 29]. In our study, immune cell type analysis showed that the immune microenvironment of non-responsive (IR-) tumors was “cold” compared to that of immune-responsive (IR+) OPSCCs. IR+ tumors showed increased levels of several immune-stimulating myeloid subsets (i.e. dendritic cells and M1 macrophages) and effector lymphoid subsets (i.e. effector memory CD8^+^ T cells and Th1), thus linking quality of innate and adaptive intratumoral responses to treatment benefit (Fig. 1d). Together, these observations suggest that modulation of the tumor immune microenvironment to an immune responsive phenotype could increase the efficacy of standard-of-care CRT treatment.

### CTX/LNIL immunomodulation reconditions the unfavorable tumor immune microenvironment

To activate the tumor immune microenvironment, we utilized an immunomodulatory treatment previously developed by our group [20] combining weekly CTX injections with continuous delivery of the iNOS inhibitor, L-NIL, in drinking water (Additional file 1: Figure S1A). To first characterize the immunomodulatory effects of the CTX/LNIL regimen in murine tumors, gene expression analysis was used to study the effects of each component of the treatment. Principal component analysis (PCA) revealed aggregate differences in gene expression and clustering based on treatment component (Additional file 1: Figure S1B; PC1 explained 54.13% and PC2 explained 14.25% of the observed variance). Immune gene-set analysis further revealed a “cold to hot” immunologic transition of the tumor following CTX and CTX/LNIL treatments (Additional file 1: Figure S1C). This dataset and existing literature [23, 35] support the notion that CTX treatment induces favorable immunomodulatory effects. Nevertheless, its combination with L-NIL further enhances immune activation and favors a unique immunologic gene-set upregulation including STING, innate response, and Th1. Further immune cell type enrichment analysis revealed gene signatures consistent with increases in favorable myeloid and lymphoid subsets following CTX and CTX/LNIL treatment (Additional file 1: Figure S1D). Interestingly, CTX/LNIL promoted significant enrichment of effector CD8^+^ T cells and Th1 cells compared to CTX alone, consistent with the infiltrates found in immune responding HPV16^+^ OPSCC human tumors (Fig. 1d). Together, these observations showed that the combination of CTX and L-NIL functions as a potent immunomodulatory regimen capable of multifactorial enhancement of the intratumoral immune microenvironment.

### Immune-suppressive effects of CRT are reversed by CTX/LNIL immunomodulation

Given the potent immunomodulatory effects induced by CTX/LNIL treatment, we hypothesized that this combination could improve the tumor immune microenvironment, rendering HPV-HNSCC tumors more responsive to CRT. Thus, we developed a combination treatment regimen in which mice bearing established mEER tumors were treated with concurrent CRT and CTX/L-NIL immunomodulation and we profiled the tumors for gene expression changes after the first week of treatment (Fig. 1e). PCA analysis revealed unique clustering of the combinatory CRT + CTX/L-NIL from that of CRT alone, but a similar clustering pattern is noted in comparison of CTX/LNIL and CRT + CTX/LNIL (Additional file 1: Figure S2A; PC1 explained 43.87% and PC2 explained 18.22% of the observed variance), revealing that CTX/LNIL further modulates the CRT-induced tumor immune microenvironment. These data are further supported by immune gene-set analysis showing that numerous anti-tumor immune response pathways downregulated by CRT were upregulated by CRT + CTX/L-NIL, including MHC, toll-like receptor (TLR), complement system, antigen processing, DC and T cell functions (Fig. 1f; for individual sample heat map see Additional file 1: Figure S2B). Interestingly, several immunosuppressive pathways (i.e. iNOS, hypoxia-adenosine and immune check points) but also the immunogenic cell death (ICD) pathway that were upregulated by CRT strongly decreased in expression after the combinatory treatment. Additional immune cell type enrichment analyses highlighted a significant increase in several innate immune cell gene subsets following the combinatory CRT + CTX/L-NIL treatment; however, only inflammatory and plasmacytoid DCs were significantly upregulated compared to CRT alone (Fig. 1g). Overall these results show that, at the level of gene expression, CTX/L-NIL enhances favorable and suppresses unfavorable intratumoral immunologic effects of CRT.

### CRT combined with CTX/L-NIL induces durable control of established tumors

**Fig. 2:**
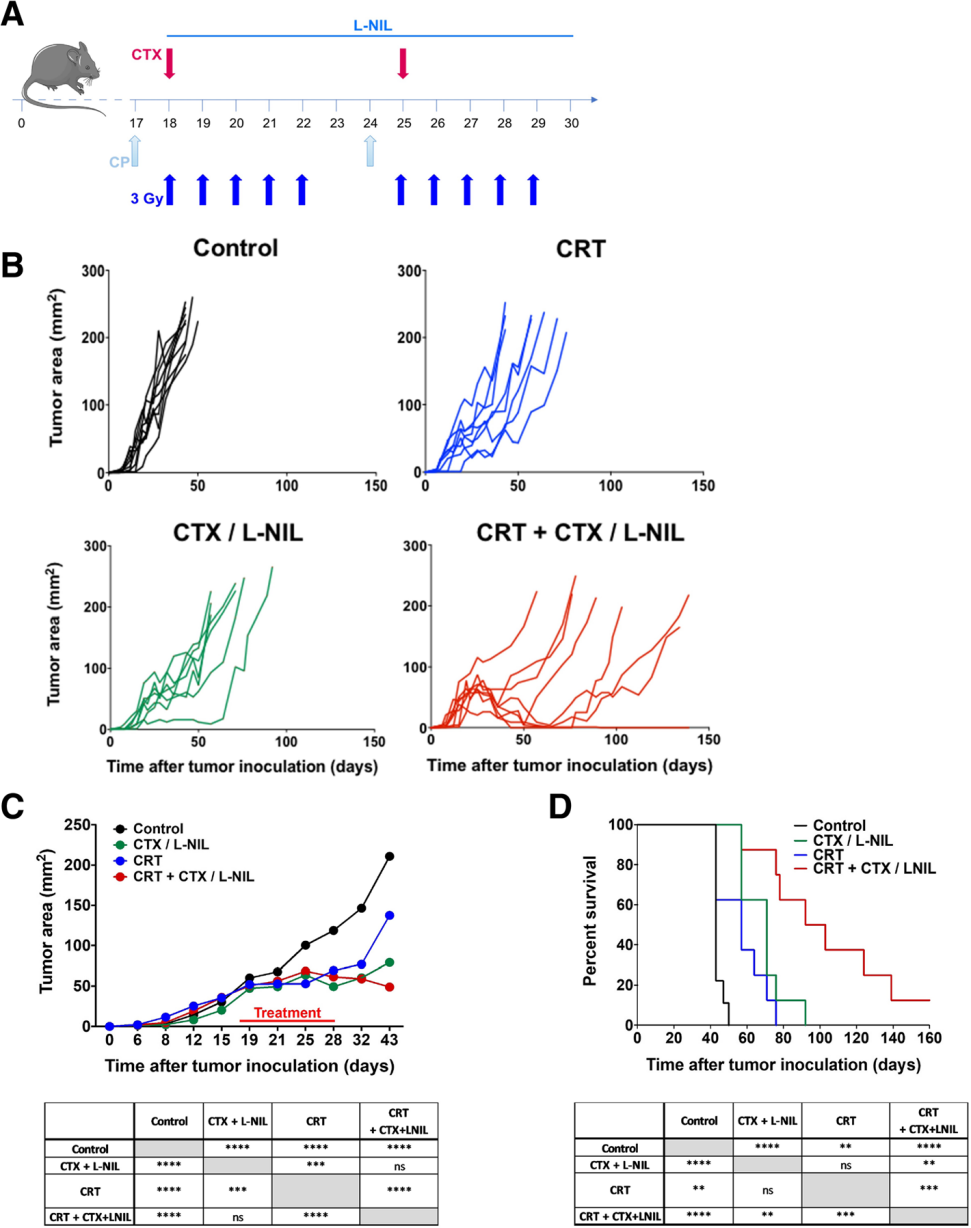
CTX/L-NIL combines with CRT to induce durable control of established tumors. Mice bearing established mEER tumors were treated with CRT (10 X 3Gy tumor-directed radiation and 83 μg/mouse weekly cisplatin i.p.) and/or CTX/L-NIL immunomodulation (CTX at 2 mg/mouse i.p. and L-NIL at 0.2% in drinking water) according to the schedule in (**a**); mice were euthanized when tumors reached 225 mm^2^. **b** Individual tumor growth curves shown by treatment group, with each mouse represented as a single line. **c** Average tumor area (top) and statistical comparison at time of first euthanasia (bottom; Tukey’s multiple comparison test). **d** Survival curves (top) and statistical comparison between treatments (bottom; Log-rank test). (**b**-**d**; N = 1 representative of 2; *n* = 8–9/group). **p < 0.01; ***p < 0.001; ****p < 0.0001; ns, not significant

To determine the therapeutic benefit of CRT and/or CTX/L-NIL immunomodulation, we assessed tumor growth and survival (Fig. 2). Briefly, mice bearing established mEER tumors were treated with CRT and/or CTX/L-NIL over a period of two weeks and were then continually monitored for long-term survival (Fig. 2a). While CRT and CTX/LNIL treatments alone each induced modest tumor growth delays compared to untreated mice, neither promoted complete tumor clearance. The combination of CRT + CTX/L-NIL significantly delayed tumor growth compared to singlet treatments and induced an average of 21% complete tumor clearance (12.5 to 30% depending on the experiment; Fig. 2b to d). To further validate these findings and determine if the treatment benefit was restricted to HPV-positive tumors, which bear viral antigens and typically have a better response to CRT, we next assessed the effects of combinatory treatments in an aggressive HPV-negative HNSCC cancer model, MOC2 [36] (Additional file 1: Figure S3). MOC2 cells were injected subcutaneously in the flank of mice and treatment was initiated once tumors became established (approximately 19 mm^2^ in size; Additional file 1: Figure S3A). Combinatory treatment significantly delayed tumor growth compared to CRT and CTX/LNIL alone (Additional file 1: Figure S3 B and C) and increased overall survival by 23 days compared to conventional CRT (Additional file 1: Figure S3D). Collectively, this dataset shows that CTX/LNIL immunomodulation enhances the treatment benefit of conventional CRT regardless of HPV-status, suggesting that this therapeutic approach may be applicable to treatment of a variety of solid tumors.

### CTX/L-NIL favorably reprograms the myeloid tumor microenvironment in CRT treated tumors

**Fig. 3:**
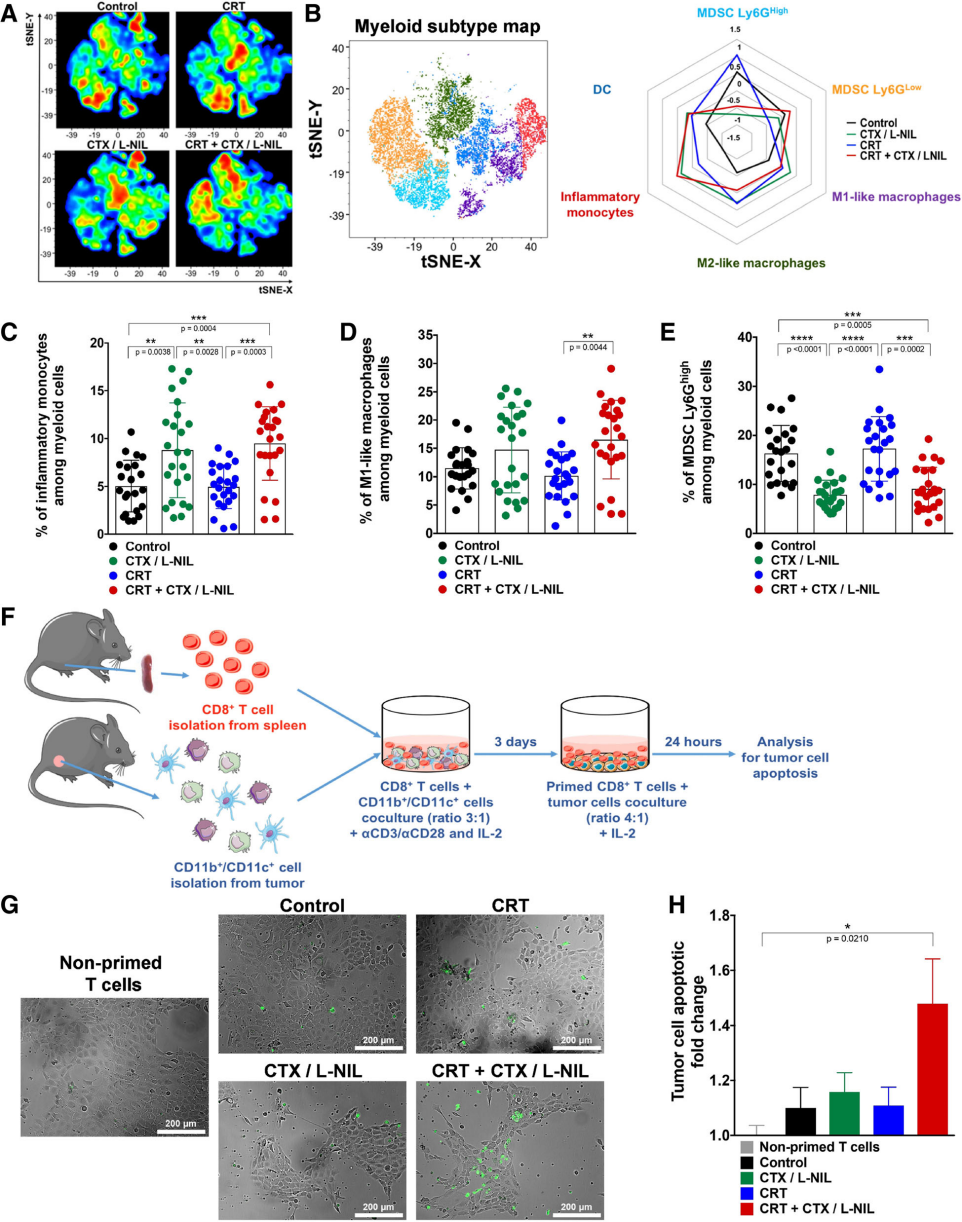
CRT + CTX/L-NIL reprograms the myeloid tumor microenvironment. Established mEER tumors were treated with CRT and/or CTX/L-NIL and harvested after the first week of treatment, as shown in to Fig. 1e, and tumor myeloid cell infiltrate was analyzed using flow cytometry (**a**-**e**; see Additional file 1: Figure S4 for myeloid flow cytometry gating strategy) and ex vivo co-culture (**f**-**h**). **a** Myeloid-focused tSNE (among CD11b^+^/CD11c^+^ cells) from flow cytometry data for each treatment group. **b** Corresponding tSNE color-map (left) and radar plot (right) showing myeloid sub-type alterations between each treatment group as z-scores (myeloid sub-type color in radar plot corresponds with their color in tSNE map; *N* = 1 representative of 3; n = 8–9/group). **c-e** Percentage of myeloid sub-types among total myeloid cell tumor infiltrate (CD11b^+^/CD11c^+^), including inflammatory monocytes **(c)**, M1-like macrophages **(d**), and granulocytic MDSCs **(e)** (*N* = 3; *n* = 23–25/group; Tukey’s multiple comparison test for inflammatory monocytes and Dunn’s multiple comparison test for M1-like macrophages and granulocytic MDSCs). **f** Experimental schematic used to test treatment-induced myeloid influence on CD8^+^ T cell cytotoxicity. Naïve splenic CD8^+^ T cells were stimulated with anti-CD3/CD28 antibodies and IL-2 in the absence or presence of myeloid cells (CD11b^+^ and CD11c^+^ cells) isolated from tumors that received different treatments for 3 days and then co-cocultured with mEER cells in presence of IL-2. After 24 h, apoptotic tumor cells were detected by Annexin V/ PI staining. **g** Representative images of mEER tumor cells stained for Annexin V (shown in green) following co-culture (scale bars show 200 μm). **h** mEER tumor cell apoptotic fold change (Annexin V^+^ / PI^−^) normalized to non-primed T cells (T cells not co-cultured with myeloid cells) (*N* = 4; *n* = 20–26/group; Dunn’s multiple comparison test). All bar graphs show mean +/− SD and each dot represents an individual mouse. *p < 0.05; **p < 0.01; ***p < 0.001; ****p < 0.0001

We have previously shown that CTX/L-NIL inhibits the intratumoral infiltration of immunosuppressive MDSCs [20] and our prior gene expression analysis revealed numerous anti-tumor innate gene-sets and cell types enriched following CRT + CTX/LNIL treatment compared to CRT alone (Fig. 1f and g). Overall, these observations suggest that CTX/LNIL immunomodulation may favorably alter the myeloid tumor microenvironment. Thus, using flow cytometry, we assessed the impact of CTX/LNIL and/or CRT treatment on the tumor myeloid composition and function (Fig. 3). To eliminate immunologic bias due to tumor size, we chose to isolate tumors and observe the immune response at an intermediate treatment timepoint (after one week) when tumor sizes were comparable between all groups (similar to Fig. 1e). The analysis of myeloid cell subpopulations (among CD11b^+^/ CD11c^+^ cells) was performed using flow cytometry (for myeloid gating strategy see Additional file 1: Figure S4A, as previously described in [37]).

Due to the complexity of representing multiple immune subsets comprehensively, we utilized t stochastic neighborhood embedding (tSNE) to map high dimensional data onto two dimensional graphs [38]. tSNE analysis visually demonstrates strong myeloid subtype variation correlating to treatment (Fig. 3a) and quantification of these data shown as Z-scores in the radar plot highlights this effect (Fig. 3b). Among the subpopulations assessed, percentages of both inflammatory monocytes (characterized by the expression of CD11b^+^, MHCII^low^, Ly6C^+^, CCR2^+^, CX3CR1^+^, iNOS^+^ (Additional file 1: Figure S4B)) and M1-like macrophages were increased following CTX/LNIL treatment. These two populations are further increased 2-fold and 1.6-fold, respectively, with CRT + CTX/LNIL compared to CRT alone (Fig. 3c and d). Furthermore, CRT + CTX/L-NIL significantly reduced the percentage of the immunosuppressive granulocytic MDSCs by 2-fold compared to CRT (Fig. 3e). Since both inflammatory monocytes and M1-like macrophages have been reported to support anti-tumor immune responses [39] and granulocytic MDSC are known to be highly immunosuppressive, this suggests that CRT + CTX/LNIL promotes the development of a beneficial myeloid tumor microenvironment.

Myeloid profiling was also performed in other lymphoid organs during treatment, in particular draining lymph nodes (dLNs) and spleen (Additional file 1: Figure S5). In the dLNs, CRT + CTX/L-NIL promoted a 2.8-fold decrease in granulocytic MDSCs compared to CRT (Additional file 1: Figure S5 C), however, no other notable changes among myeloid subsets were observed in dLNs and spleen (Additional file 1: Figure S5 A-H). This suggests that the effects induced by the combinatory treatment are strongly localized to the tumor microenvironment. Overall, these observations demonstrate that CTX/L-NIL immunomodulation can be combined with CRT to favorably reprogram the tumor myeloid microenvironment, increasing cell subtypes known to benefit anti-tumor immune responses (inflammatory monocytes and M1-like macrophages) and decreasing immunosuppressive cell subsets (granulocytic MDSCs).

### Improved myeloid tumor microenvironment facilitates CD8^+^ T cell cytotoxic response

Since myeloid cells can both promote and suppress T cells according to their subtype and activation status [40], we sought to determine whether the favorable shift in the myeloid microenvironment induced by combinatory treatment influenced T cell anti-tumor cytotoxicity. To assess this, we performed an ex vivo CD8^+^ T cell cytotoxic assay in which naïve splenic CD8^+^ T cells were stimulated (using anti-CD3, anti-CD28, and IL-2) in the presence of myeloid cells (CD11b^+^ and CD11c^+^ cells) isolated from treated tumors (as previously described in [32]). The myeloid-primed and stimulated CD8^+^ T cells were then co-cultured with mEER tumor cells and assessed for T cell induced cytotoxicity using Annexin-V/PI staining (Fig. 3f). Microscopic examination of Annexin V showed an increase in tumor cell apoptosis after CRT + CTX/L-NIL treatment compared to single therapies (Fig. 3g). Quantitative flow cytometry analysis revealed a significant improvement in the CD8^+^ T cell cytotoxicity, compared to non-primed stimulated CD8^+^ T cells, when influenced by the CRT + CTX/L-NIL intratumoral myeloid compartment (Fig. 3h). These data suggest that the favorable myeloid shift induced with the combination of CRT and CTX/LNIL lends to enhanced CD8^+^ T cell cytotoxicity.

### CTX/LNIL improves the T cell tumor microenvironment in CRT treated tumors

**Fig. 4:**
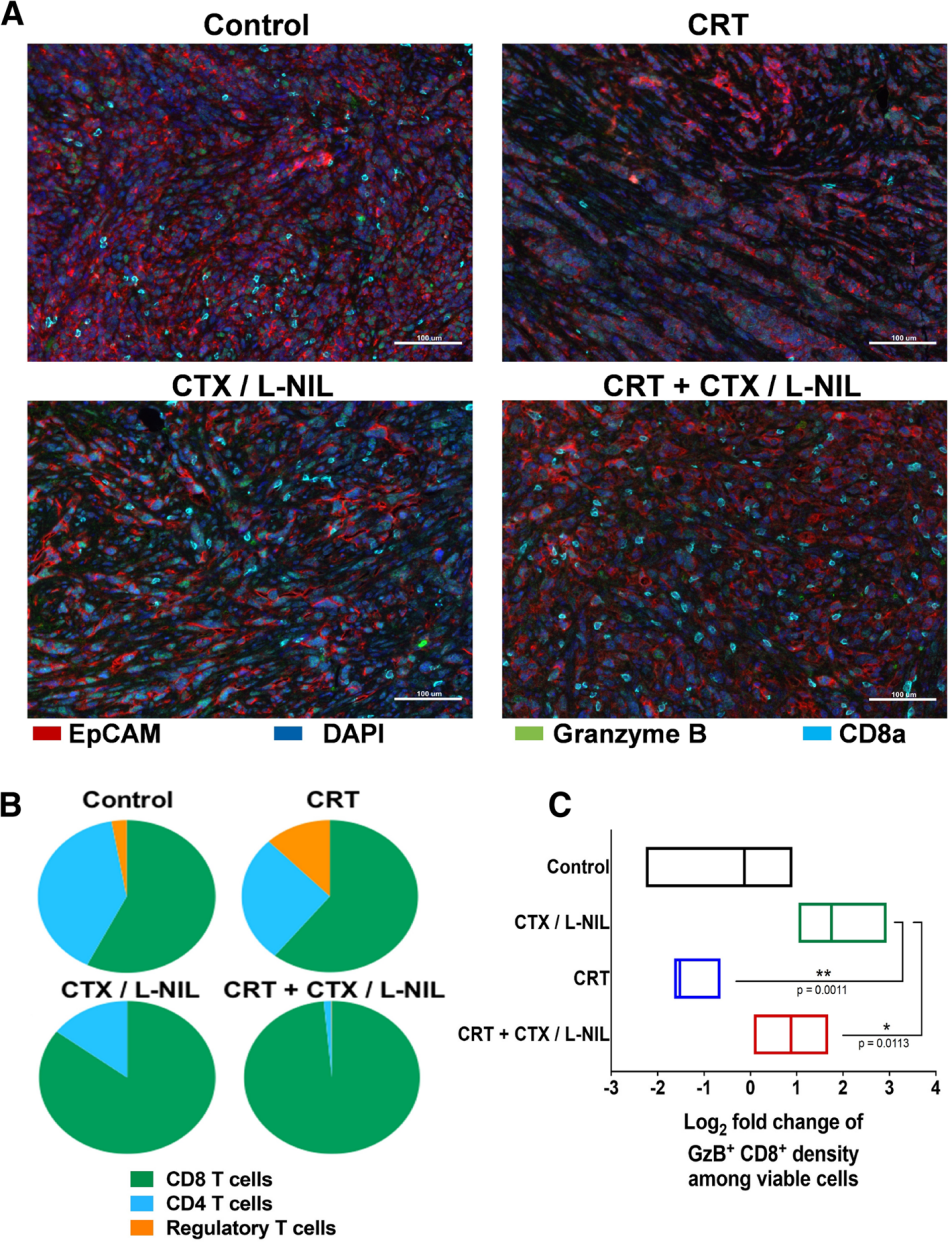
CRT + CTX/L-NIL promotes intratumoral CD8^+^ T cell infiltration and activation. Established mEER tumors were treated with CRT and/or CTX/L-NIL and harvested after the first week of treatment, as shown in Fig. 1e, and tumor lymphocyte infiltrate was analyzed using quantitative multiplex immunofluorescence (qmIF; **a**-**c**). **a** Representative multiplex images of mEER tumors showing DAPI (nuclei, dark blue), EpCAM (tumor, red), CD8α (CD8^+^ T cells, cyan), and Granzyme B (T cell cytotoxic marker, green). **b** Pie-charts showing T cell subset fractions among total T cells for each treatment (fraction averaged across 5 images per tumor and *n* = 3/group). **c** Log_2_ fold change of Granzyme B (GzB)^+^ CD8^+^ cells normalized to control group (N = 1; n = 3/group; Tukey’s multiple comparison test). Bar graphs show mean +/− SD. *p < 0.05; **p < 0.01

**Fig. 5:**
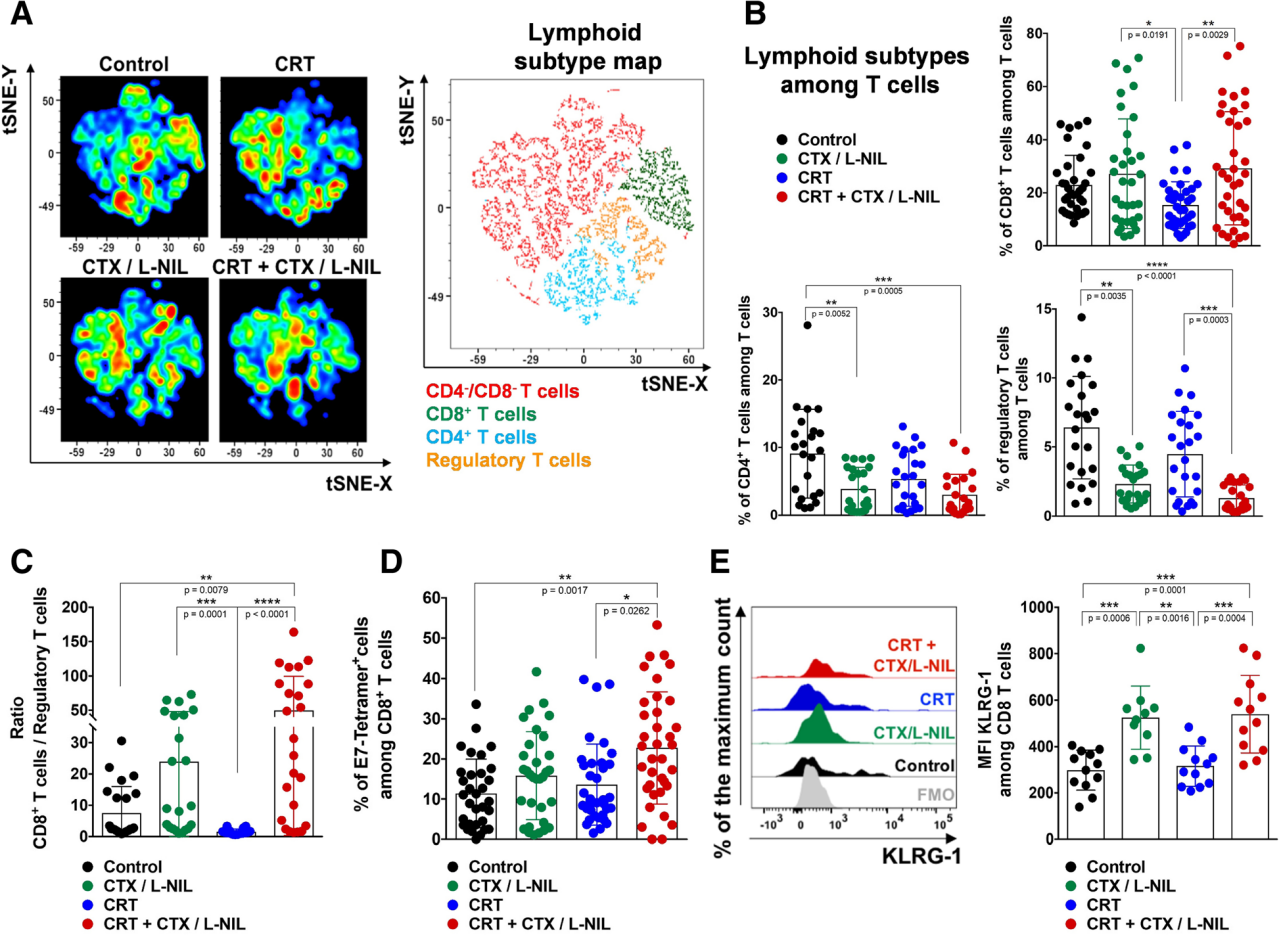
CRT + CTX/L-NIL enhances the lymphoid tumor microenvironment. Established mEER tumors treated with CRT and/or CTX/L-NIL and were harvested similar to Fig. 1e, and tumor lymphocyte infiltrate was assessed using flow cytometry (**a**-**e**; see Additional file 1: Figure S6 for lymphocyte flow cytometry gating strategy). **a** Lymphoid-focused tSNE (among TCRβ^+^ cells) from flow cytometry data for each group of treatment (left) and corresponding color-map (right) with colored lymphocyte subsets listed below (N = 1 representative of 5; n = 8–9/group). **b** Percentage of lymphoid sub-types among total tumor infiltrating T cells (TCRβ^+^ cells), including CD8^+^ T cells (top right), CD4^+^ T cells (bottom left) and regulatory T cells (bottom right) (N = 3–4; n = 23–36/group; Tukey’s multiple comparison test for CD8^+^ T cells and Dunn’s multiple comparison test for CD4^+^ and regulatory T cells). **c** Ratio of CD8^+^ T cells/ regulatory T cells percentages (N = 3; n = 23–24/group; Dunn’s multiple comparison test). **d** Percentage of E7-Tetramer^+^ among CD8^+^ T cells (*N* = 5; *n* = 33–37/group; Dunn’s multiple comparison test). **e** Representative flow cytometry histograms showing KLRG-1 expression among CD8^+^ T cells expressed as the % of the maximum count (left) and cumulative median fluorescence intensity (MFI) for each treatment group (right). FMO (Fluorescence minus one) is a mixture of all antibodies permitting CD8^+^ T cell identification without the phenotypical marker of interest (*N* = 2, *n* = 12; Tukey’s multiple comparison test). All bar graphs show mean +/− SD and each dot represents an individual mouse. *p < 0.05; **p < 0.01; ***p < 0.001; ****p < 0.0001

Based on the above data and our previous report showing that CTX/LNIL immunomodulation promotes CD8^+^ T cell tumor infiltration [20], we assessed whether CTX/LNIL and/or CRT could favorably alter the intratumoral lymphoid compartment using multi-color immunofluorescence and flow cytometry (Figs. 4 and 5). After one week of treatment (similar to Fig. 1e) we profiled tumors by multiplex immunofluorescence [31]. We observed an increase in CD8^+^ T cell tumor infiltration and a higher level of cytoplasmic Granzyme B after CTX/L-NIL and combinatory treatment (Fig. 4a). Image quantification of T cell subsets further revealed that CTX/LNIL immunomodulation promoted a CD8^+^ T cell dominated tumor and a drastic depletion of Tregs, and this effect was maintained when combined with CRT (Fig. 4b). Additionally, we observed that CTX/LNIL improved CD8^+^ T cell activation compared to CRT, as evidenced by the expression of cytoplasmic Granzyme B. When combined, although non-significantly, CRT + CTX/L-NIL induces a 4-fold increase in the intratumoral density of CD8^+^ T cells expressing Granzyme B compared to CRT alone (Fig. 4c).

To further characterize the influence of combinatory treatment on changes within the T cell tumor microenvironment, we performed flow cytometry (for lymphocyte gating strategy see Additional file 1: Figure S6A). tSNE analysis revealed qualitative shifts of intratumoral T cell subsets correlating to treatment (Fig. 5a). Further quantification revealed a 1.8-fold increase in the percentage of CD8^+^ T cells and a 3.4-fold decrease in Tregs after the combinatory CRT + CTX/LNIL treatment compared to CRT alone (Fig. 5b). These changes resulted in a 31.8-fold increase in the CD8^+^ T cell/Treg ratio after CRT + CTX/L-NIL compared to CRT alone (Fig. 5c). Further T cell subset analysis in other lymphoid organs demonstrated that the combinatory CRT + CTX/L-NIL treatment also reduced the percentage of Tregs in the dLNs (Additional file 1: Figure S7C) but not in the spleen (Additional file 1: Figure S7G) compared to CRT alone. The percentage of CD8^+^ and CD4^+^ T cells in dLNs (Additional file 1: Figure S7A and B) and spleen (Additional file 1: Figure S7E and F) were unchanged and thus the ratio of CD8^+^ T cells / Tregs was only significantly increased in dLNs but not in the spleen after CRT + CTX/L-NIL therapy compared to CRT alone (Additional file 1: Figure S7 D and H). Overall these data suggest that the combination of CRT and CTX/LNIL strongly and specifically activates the intratumoral T cell microenvironment.

### CRT + CTX/L-NIL changes the phenotype of tumor-infiltrating CD8^+^ T cells

To further understand the consequences of the improved CD8^+^ T cell infiltration induced by the combinatory treatment, we next analyzed the tumor-infiltrating CD8^+^ T cells for various functional markers. We first tested whether CRT + CTX/L-NIL could improve CD8^+^ T cell tumor specificity by evaluating E7-specific tetramers binding by flow cytometry (Additional file 1: Figure S6B). This revealed that the proportion of intratumoral CD8^+^ T cells specific for the E7 antigen expressed by mEER tumor cells was significantly increased following CRT + CTX/LNIL treatment (more than 22.7% of CD8^+^ T cells on average) (Fig. 5d). Based on a consensus nomenclature defined for murine and human CD8^+^ T cell phenotypes [41], we monitored the expression of several markers associated with T cell effector, memory, and exhaustion functions using flow cytometry (Fig. 5e and Additional file 1: Figure S8). CRT + CTX/L-NIL promoted a significant increase in the expression of killer cell lectin-like receptor G1 (KLRG1), a marker commonly associated with effector CD8^+^ T cell subsets [42]. However, perforin expression on CD8^+^ T cells was significantly decreased after CRT + CTX/L-NIL treatment compared to CRT alone, suggesting that CD8^+^ T cells had entered a later killing phase [43]. Collectively, these observations show that the tumor infiltrating CD8^+^ T cells promoted by the combinatory CRT + CTX/LNIL treatment exhibit improved specificity and effector phenotypes compared to CRT alone.

### Tumor growth inhibition induced by CRT + CTX/LNIL is dependent on CD8^+^ T cells

**Fig. 6:**
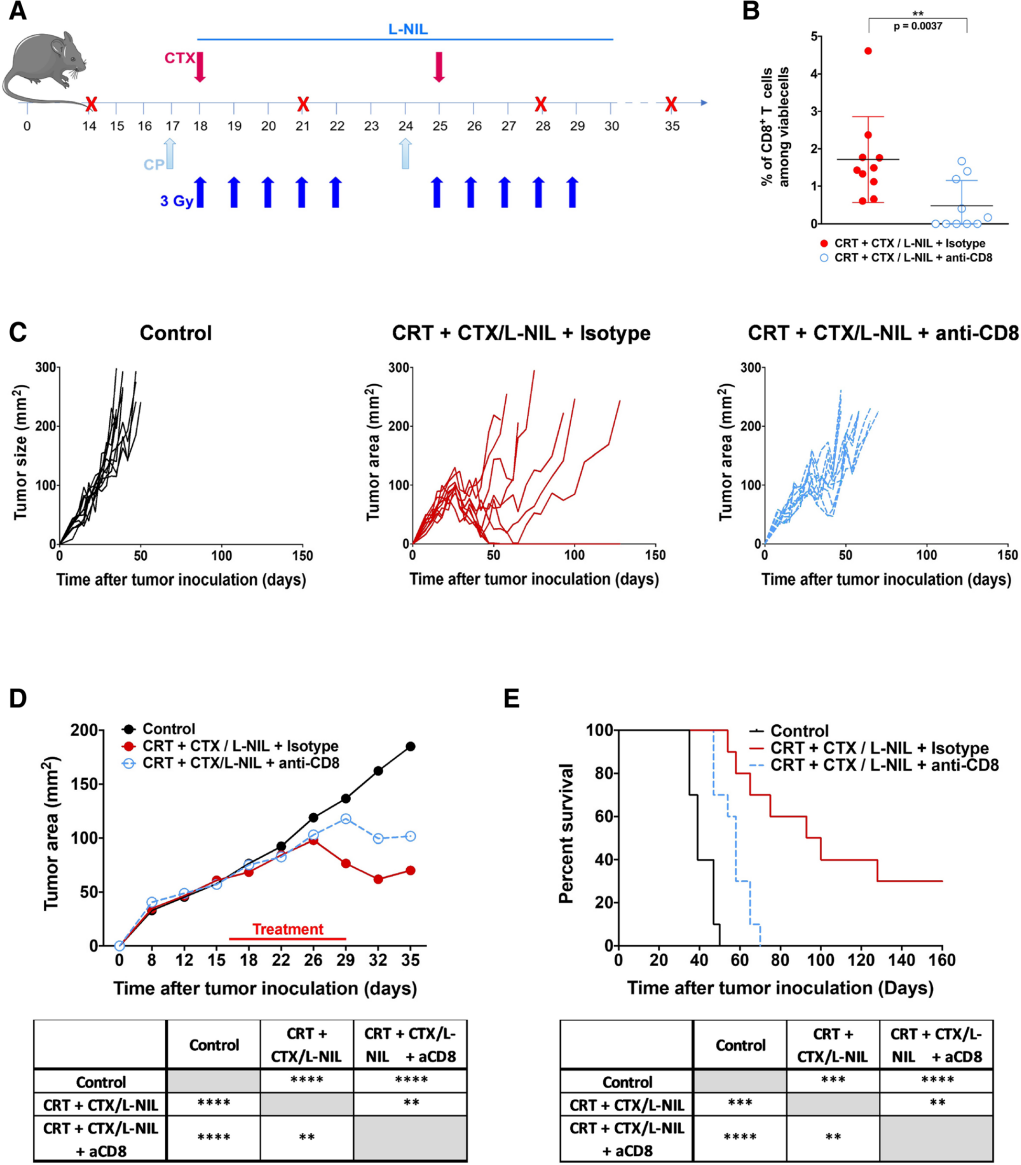
Tumor growth inhibition induced by CRT + CTX/L-NIL requires CD8^+^ T cells. Established mEER tumors were treated with CRT + CTX/L-NIL and anti-CD8α depleting antibody, or isotype control antibody, according to the schedule in (**a**); mice were euthanized when tumors reached 225 mm^2^. **b** CD8^+^ T cell percentages among total viable cells in the blood at day 29 of treatment as assessed by flow cytometry (*N* = 1; *n* = 10/group, each as an individual dot; data are means +/− SD; Mann-Whitney test). **c** Individual tumor growth curves shown by treatment group, with each mouse represented as a single line. **d** Average tumor area (top) and statistical comparison at time of first euthanasia (bottom; Tukey’s multiple comparison test). **e** Survival curves (top) and statistical comparison between treatments (bottom; Log-rank test). (**c**-**e**; *N* = 1 representative of 2; *n* = 10/group). *p < 0.05; **p < 0.01; ***p < 0.001; ****p < 0.0001; ns, not significant

Since our data show that CTX/L-NIL immunomodulation enhances susceptibility of immune-refractory tumors to CRT by changing the myeloid compartment, leading to enhancements in CD8^+^ T cell specificity and function, we examined whether improved tumor regression and survival was dependent on CD8^+^ T cells (Fig. 6). To accomplish this, depleting anti-mouse CD8 antibodies were injected two days before treatment and then weekly over the course of CRT + CTX/LNIL treatment (Fig. 6a). Flow cytometry analysis validated the depletion of CD8^+^ T cells observed in the blood (Fig. 6b). In the absence of CD8^+^ T cells, the combinatory treatment induced a small tumor growth delay, compared to control tumors (Fig. 6c and d). The stall in tumor growth observed in the absence of CD8^+^ T cells is likely a combination of direct tumor cell killing by the chemotherapeutic and radiation components of the combination treatment and non-CD8-dependent cytotoxic mechanisms (e.g. Natural Killer-mediated killing). Nevertheless, long term survival benefits are entirely lost in the absence of CD8^+^ T cells (Fig. 6e). These results confirm that CD8^+^ T cells are critical component of the therapeutic benefit induced by the combinatory CRT + CTX/LNIL treatment.

## Discussion

Enhancing the efficacy of standard-of-care therapies, such as CRT, is a critical goal in the field of oncology. In HPV-associated HNSCC, treatment response has been correlated with tumor immune cell infiltration [5]. Additionally, CRT induces immunosuppressive effects which potentially limit its effectiveness [12, 13]. Thus, we hypothesized that therapeutic outcome of CRT could be improved through favorable modulation of the tumor immune microenvironment.

The multi-faceted immunosuppressive tumor microenvironment contains numerous potential therapeutic targets whose modulation could render the tumor microenvironment more immunologically favorable. Indeed, HNSCC tumors express various immunosuppressive cytokines such as *transforming growth factor beta (*TGF-β), IL-6 or IL-10 [44, 45] and enzymes which deprive the tumor microenvironment of essential nutrients for T cell function, such as indoleamine 2,3- dioxygenase (IDO)-mediated degradation of the amino acid tryptophan [46]. Several IDO inhibitors are currently in phase I/II clinical trials [47] and may constitute an effective immunomodulatory therapy to combine with CRT. Additionally, MDSCs were found to be present in high numbers in HNSCC tumors [48] and their enzymes, arginase-1 and iNOS, are known to drive immunosuppression partially by inactivating effector T cells [49, 50]. Small molecule inhibitors have been developed to target arginase-1 and iNOS, including nor-NOHA and L-NIL, respectively [51]. iNOS, in particular, has been shown to be overexpressed in many different solid tumors and implicated in both immunosuppressive responses and resistance to chemotherapy and radiotherapy [52, 53], which support the rationale for combining an iNOS inhibitor with standard-of-care CRT. We have previously shown that while iNOS inhibition using L-NIL can inhibit MDSC recruitment to the tumor, this compound also drives a compensatory increase in Treg infiltration. This is addressed through a combination immunomodulatory approach combining L-NIL with CTX, which promotes significant antitumor immune effects by depleting intratumoral MDSCs and Tregs, respectively [20]. Herein, our ultimate goal was to determine if CTX/L-NIL immunomodulation could reverse CRT-induced immunosuppressive effects and enhance antitumor effector responses, thus maximizing its treatment efficacy.

Toward this end, we developed a clinically relevant treatment model based on standard-of-care CRT administrated to patients with HPV-HNSCC. In this model, we treated the syngeneic HPV-HNSCC mEER tumor line with fractionated (10 fractions of 3 Gy) tumor-directed radiation regimen and weekly cisplatin administration. This regimen is particularly important because different radiation schedules can induce drastically distinct immunologic responses and may even suppress immunity [14, 54]. Much of the preclinical literature studying CRT effects uses “hypo-fractionated” (small number of large doses) RT treatment schedules and shows a delay in tumor growth as well as an increased infiltration of innate and adaptive cells, such as DCs and CD8^+^ T cells in the tumor [55]. However, the infiltration of anti-tumor immune cells following hypo-fractionated radiation has also been shown to be temporally restricted and to occur primarily between 5 to 10 days after the first irradiation [55, 56]. We have also assessed the tumor immune response at various time points following our more conventionally fractionated regimen of CRT and observed that the tumor infiltration of DCs, CD8^+^ and CD4^+^ T cells reached a maximum after one week of treatment (data not shown). Our observation showed that fractionated radiation presents a timely restricted immune response similar to hypo-fractionated regimens, and the date we selected for analyzing the tumor microenvironment was during this peak window of immune cell infiltration*.* In addition to the clinical relevance of our selected treatment schedule, we also chose to assess the effects of therapeutic approaches in large, established tumors. This more accurately represents the clinical scenario, in which patients often present with advanced tumors which already have well-established immunosuppressive microenvironments. Thus, our data should provide a translationally-relevant understanding of the immunologic consequences of CRT as well as actionable therapeutic strategies for overcoming the immunological barriers.

**Fig. 7:**
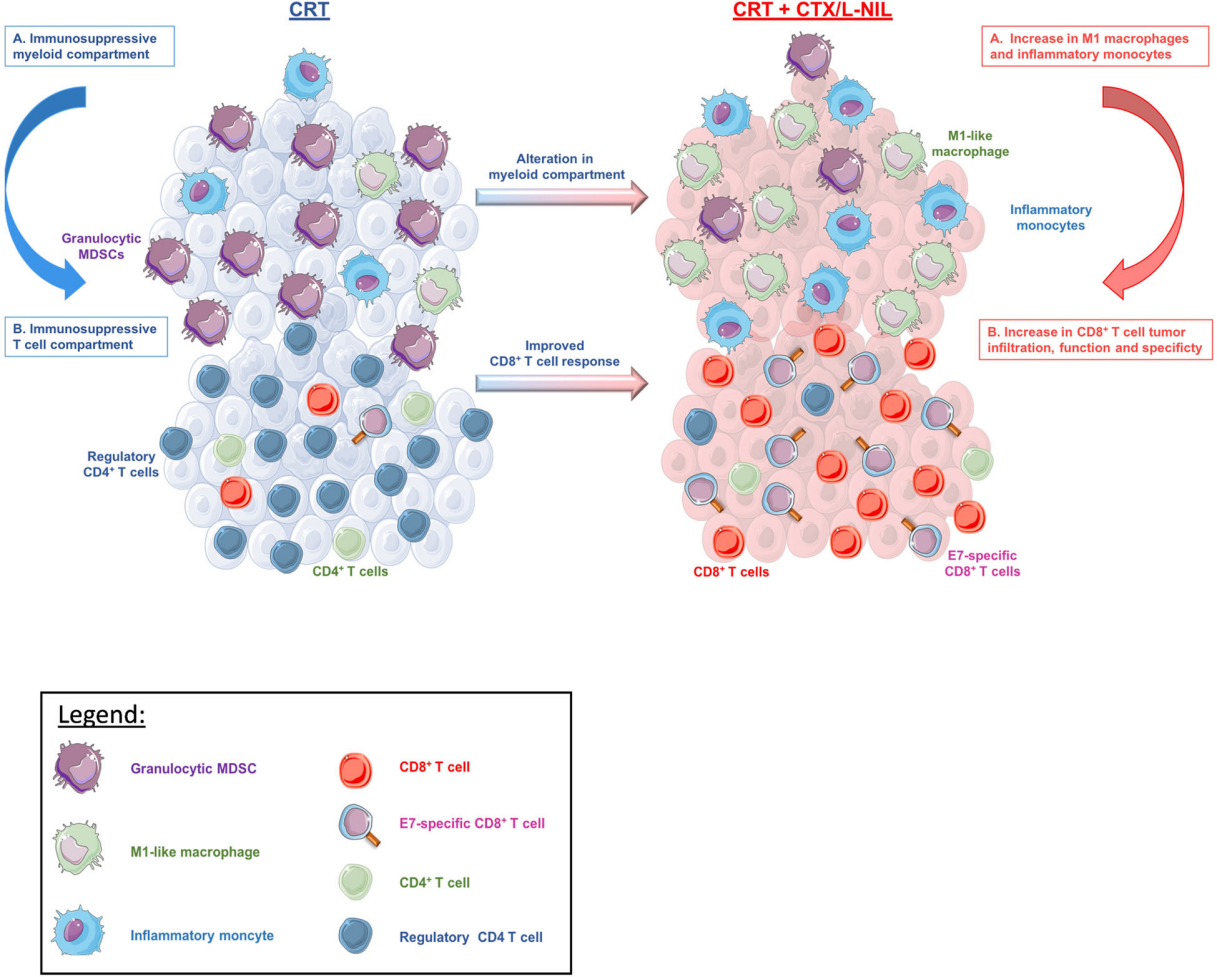
CTX/L-NIL immunomodulation renders the tumor microenvironment receptive to CRT. Schematic abstract: The tumor immune microenvironment (both myeloid and lymphoid compartments) remains “cold” after CRT treatment, and is characterized by the infiltration of granulocytic MDSCs and regulatory T cells. However, the combination of CRT with CTX/L-NIL reverses these immunosuppressive effects and further promotes increases in M1-like macrophages and inflammatory monocytes in the tumor. This favorable myeloid alteration likely plays a role in the improved intratumoral CD8^+^ T cell response, which shows enhanced specificity, effector and memory phenotypes

Herein, we showed that CTX/L-NIL immunomodulation combined with CRT promoted significant enhancement of treatment efficacy, with 21% complete tumor rejection in a CD8-dependent manner. In tumors of mice receiving the combinatory treatment, we observed significant changes in the myeloid immune microenvironment, including an increase in various myeloid populations known to promote anti-tumor effects (i.e. inflammatory monocytes and M1-like macrophages) and a decrease in immunosuppressive granulocytic MDSCs. This modification in the myeloid microenvironment likely contributes to an improved T cell compartment within the tumor, including an increase in the ratio CD8^+^ T cells/regulatory T cells and in the percentage of E7-specific CD8^+^ T cells compared to CRT alone. Gene expression analysis further corroborated these effects as it showed that CRT + CTX/L-NIL upregulated gene sets related to MHC, antigen processing, dendritic cell function, and induced a “hot” (activated) intratumoral immunologic state. Moreover, the analysis of tumor gene expression showed an increase in immunologic cell death (ICD) pathway compared to control group for each of singlet therapies (CRT or CTX/L-NIL), suggesting that these treatments favor ICD. Surprisingly, the ICD pathway appears to be decreased following the combination of CRT + CTX/L-NIL compared to singlet treatments, suggesting that induction of immunogenic cell death is not the major mechanism contributing to the efficacy of the combinatory treatment. Overall, our results demonstrated that CTX/LNIL immunomodulation can greatly improve the treatment effects of CRT by heating up the tumor immune microenvironment (Fig. 7), towards a similar constitution that in the clinic has been associated with a favorable response to (chemo) radiotherapy (Fig. 1d and [28]).

While CD8^+^ T cell phenotype profiling showed an increase in markers of T cell memory, tumor rechallenge experiments revealed a lack of functional immunologic memory response (data not shown). This constitutes a limitation of our results and mandates further investigation to understand and address this limitation in long-term memory recall response. A number of recent studies suggest that radiation fractionation schedules may have significant treatment implications on the anti-tumor functionality of the immune system. Previous work has shown that hypofractionated radiation schedules can increase the frequency of circulating T cells, and promote higher numbers of CD8^+^ T cells [57, 58]. Thus, future work will compare the immunologic consequences, especially those pertaining to immunologic memory, of different radiation fractionation schedules. Our work also suggests that immune microenvironment modulation could be utilized to improve treatment results of other immunotherapeutic approaches (i.e. immune checkpoint inhibitors, chimeric antigen receptor T cells, cancer vaccination strategies). This could potentially improve the memory response induced by our combination treatment, since immune checkpoint blockade with anti-CTLA-4 has been shown to enhance the generation of memory T cells in mice [59]. It is also likely that additional strategies to improve the number of HPV-E6/E7 specific CD8^+^ T cells, such as vaccination with long peptides against these antigens, could also improve the overall treatment benefit of this regimen [60].

In an effort to enhance the translatability of this work, we utilized a repurposing approach using two clinically-relevant drugs to overcome the negative effects of the tumor immune microenvironment. This combination included CTX, a widely utilized chemotherapeutic agent which is commonly used as an immunomodulator; and L-NIL, which has been tested in clinical trials for asthma and inflammatory diseases [19]. Despite the translational potential of this study, a few key issues remain to be addressed before clinical investigation of the CTX/LNIL immunomodulatory regimen. The first relates to the injection timing and the dose of CTX used, as this can have major influences on treatment response. Contrary to high dose CTX, which is lymphodepleting and cytotoxic, low dose of CTX is immune dependent, increases the anti-tumor immune response and favors T cell tumor infiltration [61]. In terms of treatment schedule, metronomic dosing has been previously tested in the clinic and was found to selectively deplete Tregs and improve overall T cell function and specificity [62, 63]. However, another study demonstrated optimal immunomodulating effects with a single injection of CTX slightly above the ablative dose [64]. Thus, a CTX dose titration and schedule-finding study would likely be necessary to establish the optimal immunomodulatory response. The second key issue relates to the iNOS inhibitor utilized in this study, L-NIL, which, despite prior testing in clinical trials for benign diseases, requires additional clinical assessment before it could be used for treatment of cancer patients. However, the efficacy of other commonly used drugs with iNOS inhibiting activity, such as doxycycline or phosphosdiesterase 5 inhibitors (sildenafil and tadalafil), could be investigated in parallel to facilitate rapid translation to clinic trials [65–67].

## Conclusion

In conclusion, our study demonstrates that immunomodulation of the tumor microenvironment can render refractory tumors susceptible to CRT. The clinical relevance of the models studied and the combination of standard-of-care CRT with repurposed immunomodulatory drugs has the potential to accelerate clinical translation of this approach.

## Additional file

Additional file 1: **Figure S1**. The combination of CTX / L-NIL reverses the cold tumor microenvironment. **Figure S2**. CTX / L-NIL activates the immune microenvironment of CRT treated tumors. **Figure S3**. CTX/L-NIL improves CRT treatment effects in established HPV-negative tumors. **Figure S4**. Gating strategy for myeloid sub-types and inflammatory monocyte phenotyping. **Figure S5**. Systemic myeloid effects induced by CRT+CTX/L-NIL. **Figure S6**. Gating strategy for lymphocyte sub-types. **Figure S7**. Systemic lymphoid effects induced by CRT+CTX/L-NIL. **Figure S8**. CD8^+^ T cell phenotype in tumors. **Table S1**. Flow cytometry antibodies used for myeloid subset analysis. **Table S2**. Flow cytometry antibodies used for lymphocyte and CD8^+^ T cell subset analysis. **Table S3**. Immune pathway signature gene list used in gene expression analysis. **Table S4**. Immune cell type signature gene list used in gene expression analysis [68–70]. (ZIP 12061 kb)

## Abbreviations

CRT: Chemoradiotherapy
CTX: Cyclophosphamide
DC: Dendritic cell
KLRG1: Killer cell lectin-like receptor
HNSCC: Head and neck squamous cell carcinoma
HPV: Human papillomavirus
ICD: Immunogenic cell death
IDO: Indoleamine 2,3- dioxygenase
IL: Interleukins
iNOS: Inducible nitric oxide synthase
IR-: Immune response negative
IR+: Immune response positive
L-NIL: L-n6-(1-iminoethyl)-lysine
MDSC: Myeloid-derived suppressor cell
MHC: Major histocompatibility complex
OPSCC: Oropharyngeal squamous cell carcinoma
PCA: Principal component analysis
s.c.: Subcutaneously
TGF-β: Transforming growth factor beta
TLR: Toll-like receptor
Treg: Regulatory T cell
tSNE: t Stochastic neighborhood embedding
