# Correction: *Tumor microenvironment modulation enhances immunologic benefit of chemoradiotherapy*

**DOI:** 10.1136/jitc-2020-0485-9corr

**Published:** 2020-03-09

Hanoteau A, Newton JM, Krupar R, *et al*. Tumor microenvironment modulation enhances immunologic benefit of chemoradiotherapy. *J Immunother Cancer* 2019;7:10. doi: 10.1186/s40425-018-0485-9.

Since the online publication of this article, the authors have noticed that the funding acknowledgement is incomplete. As indicated in the funding acknowledgement, the study was partially funded by grant CPRIT RR160027 from the Cancer Prevention & Research Institutes of Texas. BZ is a CPRIT Scholar in Cancer Research.

