# Supplementary material for: Tumor microenvironment modulation enhances immunologic benefit of chemoradiotherapy
Source: J Immunother Cancer. 2019 Jan 15;7:10. doi: 10.1186/s40425-018-0485-9 (PMC6332704; doi:10.1186/s40425-018-0485-9)
Supplement: Supplementary file 1 — Figure S1. The combination of CTX / L-NIL reverses the cold tumor microenvironment. Figure S2. CTX / L-NIL activates the immune microenvironment of CRT treated tumors. Figure S3. CTX/L-NIL improves CRT treatment effects in established HPV-negative tumors. Figure S4. Gating strategy for myeloid sub-types and inflammatory monocyte phenotyping. Figure S5. Systemic myeloid effects induced by CRT+CTX/L-NIL. Figure S6. Gating strategy for lymphocyte sub-types. Figure S7. Systemic lymphoid effects induced by CRT+CTX/L-NIL. Figure S8. CD8+ T cell phenotype in tumors. Table S1. Flow cytometry antibodies used for myeloid subset analysis. Table S2. Flow cytometry antibodies used for lymphocyte and CD8+ T cell subset analysis. Table S3. Immune pathway signature gene list used in gene expression analysis. Table S4. Immune cell type signature gene list used in gene expression analysis [68–70]. (ZIP 12061 kb) [file 40425_2018_485_MOESM1_ESM.zip › Additional File.docx]

Supplementary Materials:

Supplementary materials and methods

*Tumor-directed radiation*

Irradiation was performed on anaesthetized tumor-bearing mice using a RadSource 2000 X-ray irradiator (160 kV, 25mA) at a dose rate of 0.031 Gy/s. Each mouse was briefly confined in a plastic restrainer and tumor-directed radiation was performed using a lead shield with an opening that exposes the mouse flank allowing the tumor site to be focally irradiated (BrainTree Scientific, Inc.). Mice were not anesthetized at the time of irradiation.

*Multispectral analysis*

Briefly, five representative areas were randomly selected. These images were factored equally into the analysis for each mouse. For spectral unmixing, examples of each fluorophore are taken from single-stained slides for each antibody, as well as a representative autofluorescence spectrum from an unstained sample. Images from each of these single-stained and unstained slides were used to create a multispectral library in inForm (PerkinElmer, Hopkinton, MA) and extracted from the multispectral data using linear unmixing. Cellular and subcellular compartments were defined by a counterstain (DAPI) to define the nucleus of each cell. Cell segmentation was adjusted based on minimum DAPI signal to accurately locate all cells and minimize hyper- and hypo-segmentation below 5% of total cells (assessed manually). Cells were then phenotyped using the phenotyping feature in inForm. Approximately 25-30 representative cells for each base variable were selected to train the phenotyping algorithm: tumor (EpCAM), T cells (CD3), and other (negative for EpCAM and CD3). Last the images were scored for intensity based on each individual secondary marker for further phenotyping of CD4, CD8, FoxP3, and Granzyme B. Finally, data obtained from all representative images were compiled to yield values for each patient. Exported inForm data from all images was processed in separate software proprietary. In this software, images were combined and analyzed to concatenate variables (i.e., CD3^+^CD8^+^Granzyme B^+^) and determine density of distinct phenotypes.

*Gene expression analysis*

Gene expression profiling was performed using the NanoString nCounter® Gene Expression system. The process is as follows:

Hybridization protocol: 100 ng of total RNA were hybridized with the NanoString Technologies nCounter® Gene Expression Mouse PanCancer Immune Profiling code set containing 770 unique pairs of 35-50bp reporter probes and biotin-labeled capture probes, including internal reference controls. Overnight hybridization occurred for 17-22 hours at 65°C.

Wash protocol: Removal of excess probes with magnetic bead purification was performed on the nCounter® Prep Station (software v4.0.11.2) on the High Sensitivity assay. Briefly, the probe-mRNA structure was affinity purified by its 3’ end to remove excess reporter probes, then by its 5’ end to remove excess capture probes. Once unbound probes were washed away, the tripartite structure was bound to the streptavidin-coated cartridge by the biotin capture probe, aligned by an electric current (negative to positive), and immobilized. Photobleaching and fluorophore degradation was prevented with the addition of SlowFade.

Scan protocol: The cartridge containing immobilized samples was transferred to the nCounter® Digital Analyzer (software v3.0.1.4) and scanned at 555 field of view (FOV). An epi-fluorescent microscope and CCD camera identified sets of fluorescent spots, which were tabulated for data output. Quality control metrics were recorded using the nSolver Analysis Software v3.0.22.

Raw read counts were normalized by NanoString nSolver (version 3.0) following the manufacture instruction. Briefly, the normalization subtracted the background measured by a negative control probe set, and the geometric mean of a set of housekeeping genes was then used to scale the read count for each sample. The normalized read counts were log2-transformed and used for differential gene expression analysis by the R package “limma”[68]. Differentially expressed genes were defined using the cutoff of fold change > 2 and adjusted p-value < 0.05 (Benjamini-Hochberg procedure). Furthermore, the single sample GSEA algorithm (ssGSEA)[69] implemented in the R package “GSVA”[70] was used to transform the gene expression into pathway activity scores. Pairwise activity scores were compared by the “limma” R package and adjusted p-value (Benjamini-Hochberg procedure) less than 0.05 was set to define differential activated pathways in the comparison. All the principle component analysis (PCA) and differential analysis were performed under the R computation environment (3.4.0). For all other graphs, the activity scores obtained were converted in z-scores. The immune pathway and immune cell type analysis are based on predetermined gene lists from NanoString and previous literature (Supplementary Table S3 and S4).

Supplementary figures

**Fig. S1. The combination of CTX / L-NIL reverses the cold tumor microenvironment. (A**-**D**) Subcutaneous established mEER tumors (day 17-18 post tumor cell injection) were treated with CTX (2mg per mouse i.p.) and/ or L-NIL (0.2% in drinking water) and total tumor RNA was extracted and processed for gene expression analysis after 1 week of treatment, according to schedule in (**A**). (**B**) PCA of total RNA expression between different treatments (each dot represents an individual mouse). (**C**) Heatmap of differential immune gene-set pathway enrichment represented as z-scores between treatment groups. (**D**) Gene-set based immune cell type enrichment comparing CTX and CTX/L-NIL represented as z-scores. (**B**-**D**; N=1; n=9/group; unpaired t test). **p* < 0.05; ***p* < 0.01; ns, not significant.

**Fig. S2. CTX / L-NIL activates the immune microenvironment of CRT treated tumors.** Established mEER tumors were treated with CRT (10 X 3Gy daily tumor-directed radiation and 83 µg/mouse weekly cisplatin i.p.) and/or CTX/L-NIL immunomodulation (CTX at 2 mg/mouse i.p. and L-NIL at 0.2% in drinking water) and total tumor RNA was extracted and processed for gene expression analysis after 1 week of treatment, according to schedule in Figure 1E. (**A**) PCA of total RNA expression between different treatments (each dot represents an individual mouse). (**B**) Heatmap of differential immune gene-set pathway enrichment represented as z-scores for individual samples clustered by treatment. (N=1; n=9/group).

**Fig. S3. CTX/L-NIL improves CRT treatment effects in established HPV-negative tumors.** Mice bearing established syngeneic MOC-2 tumors were treated with CRT (10 X 3Gy daily tumor-directed radiation and 83 µg/mouse weekly cisplatin i.p.) and/or CTX/L-NIL immunomodulation (CTX at 2 mg/mouse i.p. and L-NIL at 0.2% in drinking water) according to the schedule in (**A**); mice were euthanized when tumors reached 225 mm^2^. (**B**) Individual tumor growth curves shown by treatment group, with each mouse represented as a single line. (**C**) Average tumor area until time of first euthanasia in treated groups, statistical comparison was done at day 34 (Tukey’s multiple comparison test). (**D**) Survival curves between treatment groups (Log-rank test). (**B**-**D**; N=2; n=17-19/group). ***p* < 0.01; ****p* < 0.001; *****p* < 0.0001; ns, not significant.

**Fig. S4. Gating strategy for myeloid sub-types and inflammatory monocyte phenotyping**. (**A**) Cell suspensions from spleens, draining lymph nodes and tumors were first gated using Side Scatter (SSC) and Forward Scatter (FSC), then selected for singlet population (FSC-H vs FSC-A), viable cells (LIVE/DEAD^TM^ Fixable Blue Dead Cell Stain Kit), leukocytes (CD45), myeloid cells (CD11b^+^ and/or CD11c^+^) and dendritic cells (CD11c^+^ and MHCII^+^) or myeloid derived suppressor cells (MDSC; CD11b^+^ and Ly6G^+^). Among non-MDSCs (CD11b^+^ and Ly6G^-^), cells were gated on inflammatory monocytes (MHCII^low^ and Ly6C^+^). Among non-inflammatory monocytes (Ly6C^-^), cells were gated on tumor-associated macrophages (TAMs; Ly6C^-^ and F4/80^+^). Among TAMs, cells were selected for M1-like macrophages (M1-like; MHCII^+^) and M2-like macrophages (MHCII^-^). (**B**) Representative flow cytometry histograms showing MHCII, CD11c, Ly6G, F4/80, CX3CR1, CCR2 and iNOS expression among inflammatory monocytes in tumors expressed as the % of the maximum count. FMO (Fluorescence minus one) is a mixture of all antibodies permitting inflammatory monocyte identification without the phenotypical marker of interest (N=2; n=24-39).

**Fig. S5. Systemic myeloid effects induced by CRT+CTX/L-NIL.** dLNs and spleens were harvested on the day after the first week of treatment. Myeloid subtypes were analyzed by flow cytometry according to the gating strategy shown in Fig S4. (**A-D**) Percentage of myeloid sub-types among total myeloid cell dLN infiltrate (CD11b+ and/or CD11c+), including dendritic cells (**A**), inflammatory monocytes (**B**), MDSCs (**C**), macrophages (**D**). (N=3; n=17-25/group; Tukey’s multiple comparison test for MDSCs) and Dunn’s multiple comparison test for dendritic cells, inflammatory monocytes and macrophages). (**E-H**) Percentage of myeloid sub-types among total myeloid cell splenic infiltrate (CD11b+ and/or CD11c+), including dendritic cells (**E**), inflammatory monocytes (**F**), MDSCs (**G**) and macrophages (**H**). (N=3; n=17-25/group; Tukey’s multiple comparison test for MDSCs) and Dunn’s multiple comparison test for dendritic cells, inflammatory monocytes and macrophages). All graphs show mean +/- SD and each dot represents an individual mouse. **p* < 0.05; ***p* < 0.01; ****p* < 0.001; *****p* < 0.0001.

**Fig. S6. Gating strategy for lymphocyte sub-types**. (**A**). Cell suspensions from spleens, draining lymph nodes and tumors were first gated using Side Scatter (SSC) and Forward Scatter (FSC), then selected for singlet population (FSC-H vs FSC-A), viable cells (LIVE/DEAD^TM^ Fixable Blue Dead Cell Stain Kit), leukocytes (CD45) and T cells (TcRβ^+^). Among T cells, cells were gated on CD8^+^ T cells (CD8α^+^) and CD4^+^ T cells (CD4^+^). Among CD4^+^ T cells, cells were gated Foxp3^-^ cells (described as CD4^+^ T cells in this paper) and Foxp3^+^ cells (described as regulatory T cells in this paper). (**B**) In tumors, E7-tetramer^+^ cells were gated among CD8^+^ T cell population. Flow graphs show the percentage of E7-tetramer^+^ CD8^+^ T cells for each treatment. Control staining (left plot) had all other antibodies permitting CD8^+^ T cell identification without the E7-tetramer.

**Fig. S7. Systemic lymphoid effects induced by CRT+CTX/L-NIL.** dLNs and spleens were harvested on the day after the first week of treatment. Lymphoid subtypes were analyzed by flow cytometry according to the gating strategy shown in Fig S6. (**A-C**) Percentage of dLN lymphoid sub-types among total lymphocyte dLN infiltrate (TcRβ^+^), including CD8^+^ T cells (**A**), CD4^+^ T cells (**B**), and regulatory T cells (**C**). (**D**) Ratio of CD8^+^ T cells/Regulatory T cells. (N=3; n=20-25/group; Tukey’s multiple comparison test for CD8^+^ T cells, CD4^+^ T cells and ratio CD8^+^ T cells/Regulatory T cells and Dunn’s multiple comparison test for regulatory T cells. (**E-H**) Percentage of splenic lymphoid sub-types among total lymphocyte infiltrate (TcRβ^+^), including CD8 T cells (**E**), CD4^+^ T cells (**F**), and regulatory T cells (**G**). Ratio of CD8^+^ T cells/Regulatory T cells (**H**). (N=3; n=17-25; Tukey’s multiple comparison test for CD4^+^ T cells and ratio CD8^+^ T cells/ Regulatory T cells and Dunn’s multiple comparison test for CD8^+^ T cells and regulatory T cells). All graphs show mean +/- SD and each dot represents an individual mouse. **p* < 0.05; ***p* < 0.01; ****p* < 0.001; *****p* < 0.0001.

**Fig. S8. CD8**^+^ **T cell phenotype in tumors.** Tumors were harvested on the day after the first week of indicated treatment and immune cell suspensions were analyzed by flow cytometry to observe CD8^+^ T cell phenotypes after treatment. Heatmap showing median fluorescence intensity alterations (among CD8^+^ T cells) for various markers characteristic of CD8^+^ T cell subtype, each represented as an average z-score (N=2-4; n=10-28/group; Tukey’s multiple comparison test for TCF-1/7, KLRG1, Ki67 and Perforin and Dunn’s multiple comparison test for Eomes, PD-1, and CCR7). **p* < 0.05; ***p* < 0.01; ****p* < 0.001.

Supplementary tables

Table S1. Flow cytometry antibodies used for myeloid subset analysis

Table S2. Flow cytometry antibodies used for lymphocyte and CD8^+^ T cell subset analysis

**
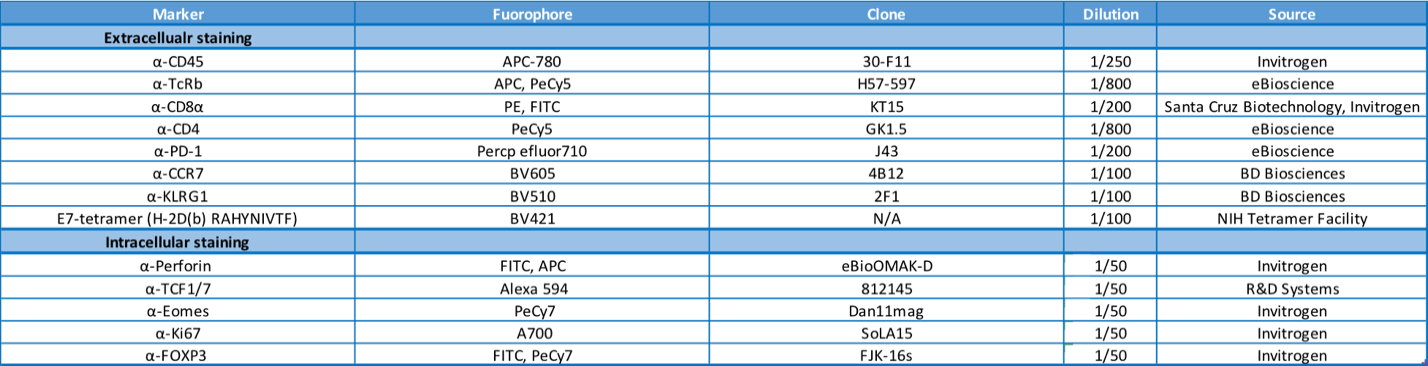
**

Table S3. Immune pathway signature gene list used in gene expression analysis


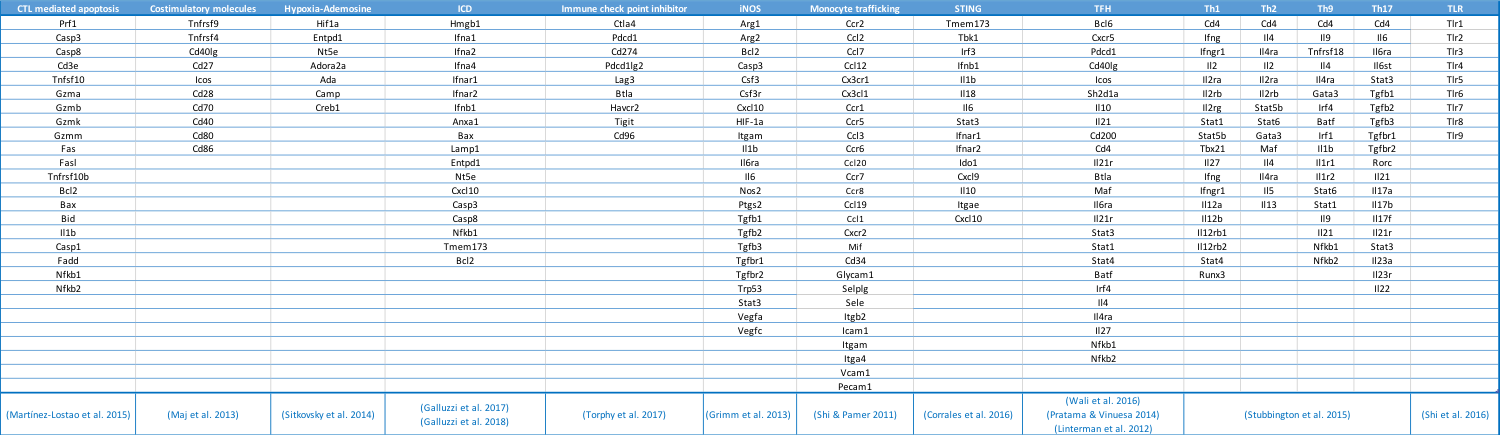


Table S4. Immune cell type signature gene list used in gene expression analysis
