# Supplementary figures and images for: Tumor microenvironment modulation enhances immunologic benefit of chemoradiotherapy
Source: J Immunother Cancer. 2019 Jan 15;7:10. doi: 10.1186/s40425-018-0485-9 (PMC6332704; doi:10.1186/s40425-018-0485-9)

## dLNs

**A**

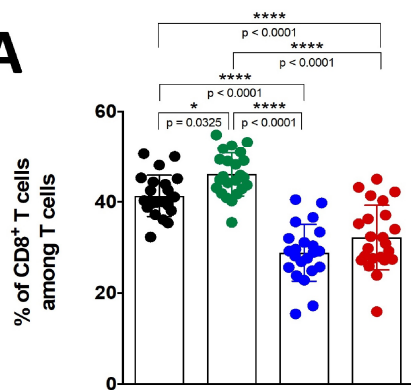

**B**

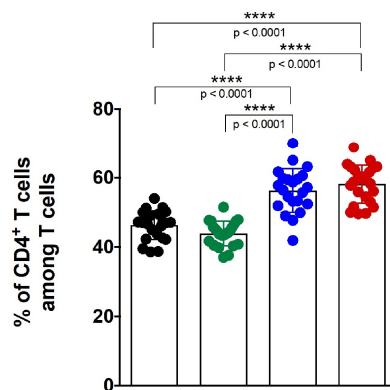

**C**

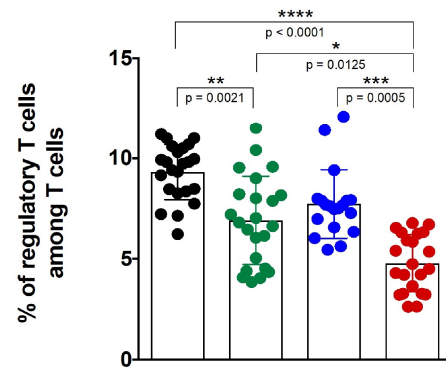

**D**

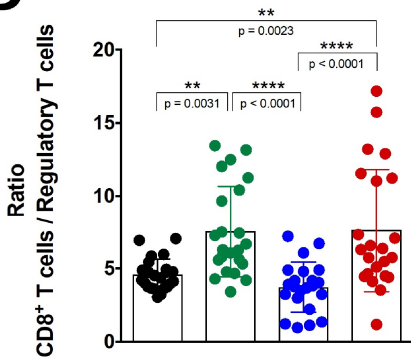

● Control  
● CTX / L-NIL  
● CRT  
● CRT + CTX / L-NIL

## Spleen

**E**

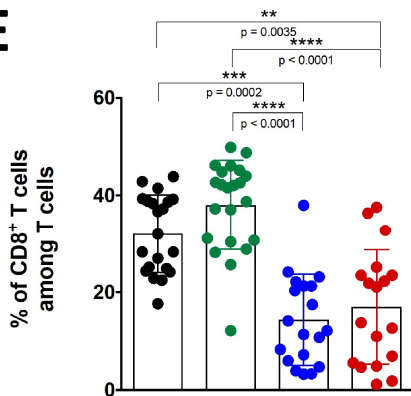

**F**

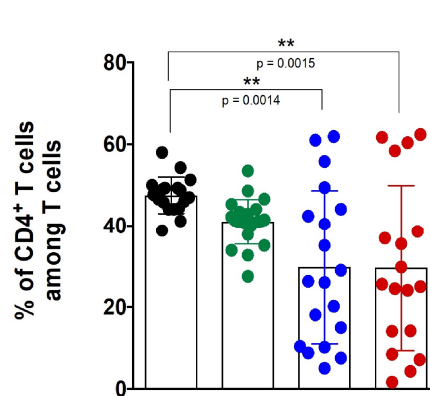

**G**

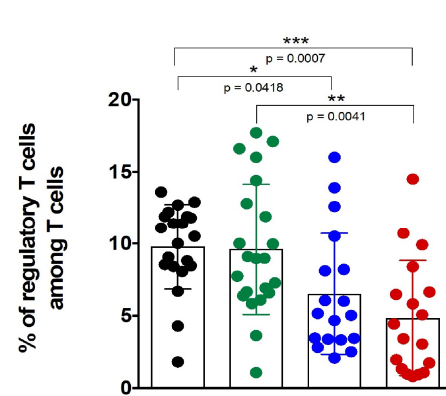

**H**

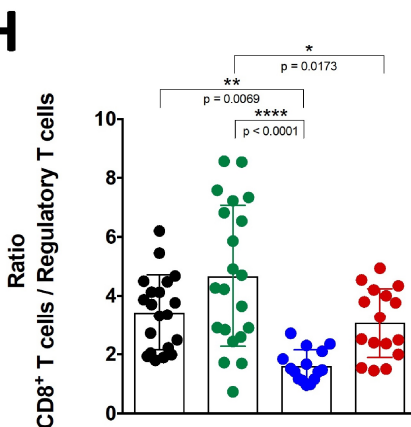

● Control  
● CTX / L-NIL  
● CRT  
● CRT + CTX / L-NIL

Supplement: Supplementary file 1 — Figure S1. The combination of CTX / L-NIL reverses the cold tumor microenvironment. Figure S2. CTX / L-NIL activates the immune microenvironment of CRT treated tumors. Figure S3. CTX/L-NIL improves CRT treatment effects in established HPV-negative tumors. Figure S4. Gating strategy for myeloid sub-types and inflammatory monocyte phenotyping. Figure S5. Systemic myeloid effects induced by CRT+CTX/L-NIL. Figure S6. Gating strategy for lymphocyte sub-types. Figure S7. Systemic lymphoid effects induced by CRT+CTX/L-NIL. Figure S8. CD8+ T cell phenotype in tumors. Table S1. Flow cytometry antibodies used for myeloid subset analysis. Table S2. Flow cytometry antibodies used for lymphocyte and CD8+ T cell subset analysis. Table S3. Immune pathway signature gene list used in gene expression analysis. Table S4. Immune cell type signature gene list used in gene expression analysis [68–70]. (ZIP 12061 kb) [file 40425_2018_485_MOESM1_ESM.zip › Fig. S7..pdf]

**A**

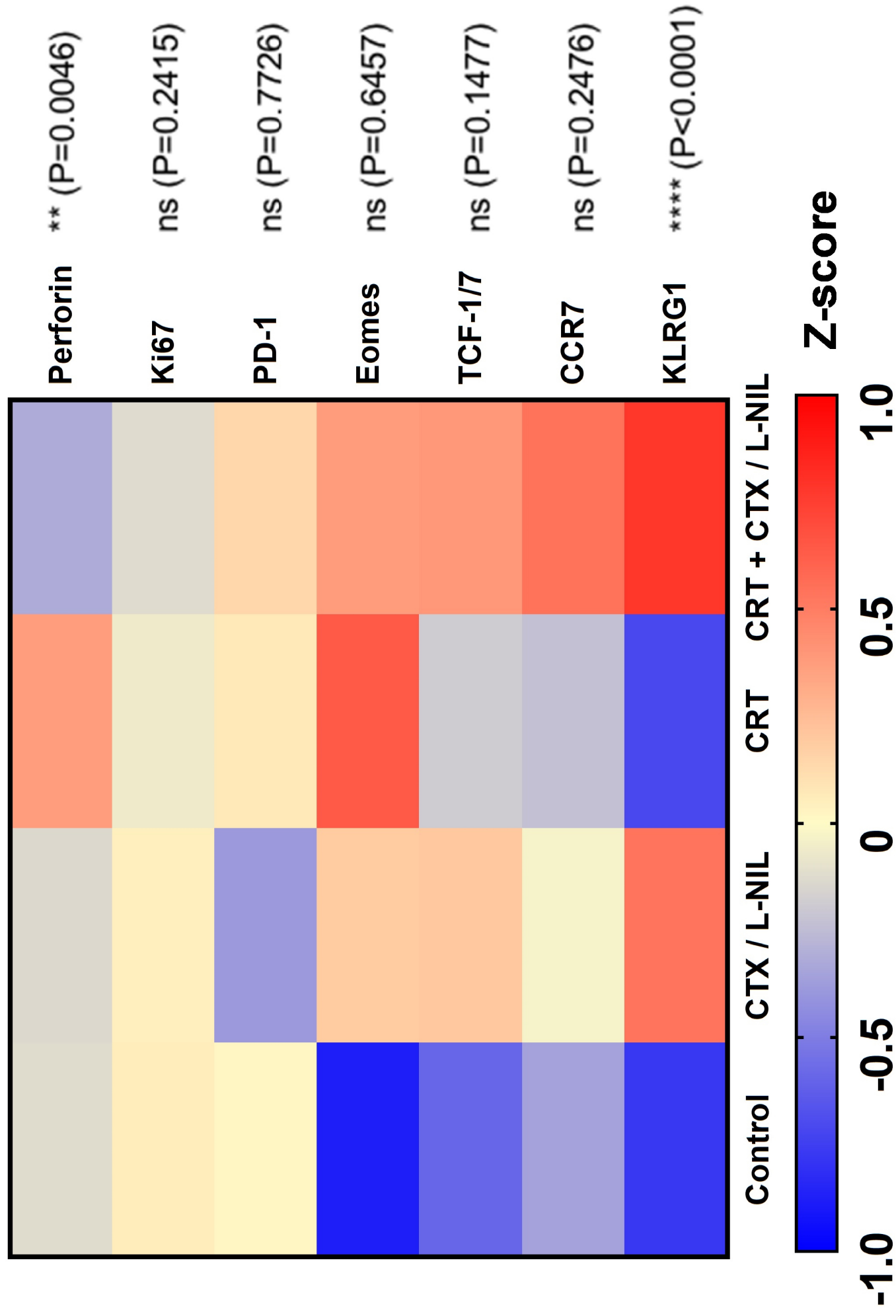

Supplement: Supplementary file 1 — Figure S1. The combination of CTX / L-NIL reverses the cold tumor microenvironment. Figure S2. CTX / L-NIL activates the immune microenvironment of CRT treated tumors. Figure S3. CTX/L-NIL improves CRT treatment effects in established HPV-negative tumors. Figure S4. Gating strategy for myeloid sub-types and inflammatory monocyte phenotyping. Figure S5. Systemic myeloid effects induced by CRT+CTX/L-NIL. Figure S6. Gating strategy for lymphocyte sub-types. Figure S7. Systemic lymphoid effects induced by CRT+CTX/L-NIL. Figure S8. CD8+ T cell phenotype in tumors. Table S1. Flow cytometry antibodies used for myeloid subset analysis. Table S2. Flow cytometry antibodies used for lymphocyte and CD8+ T cell subset analysis. Table S3. Immune pathway signature gene list used in gene expression analysis. Table S4. Immune cell type signature gene list used in gene expression analysis [68–70]. (ZIP 12061 kb) [file 40425_2018_485_MOESM1_ESM.zip › Fig. S8..pdf]

**A**

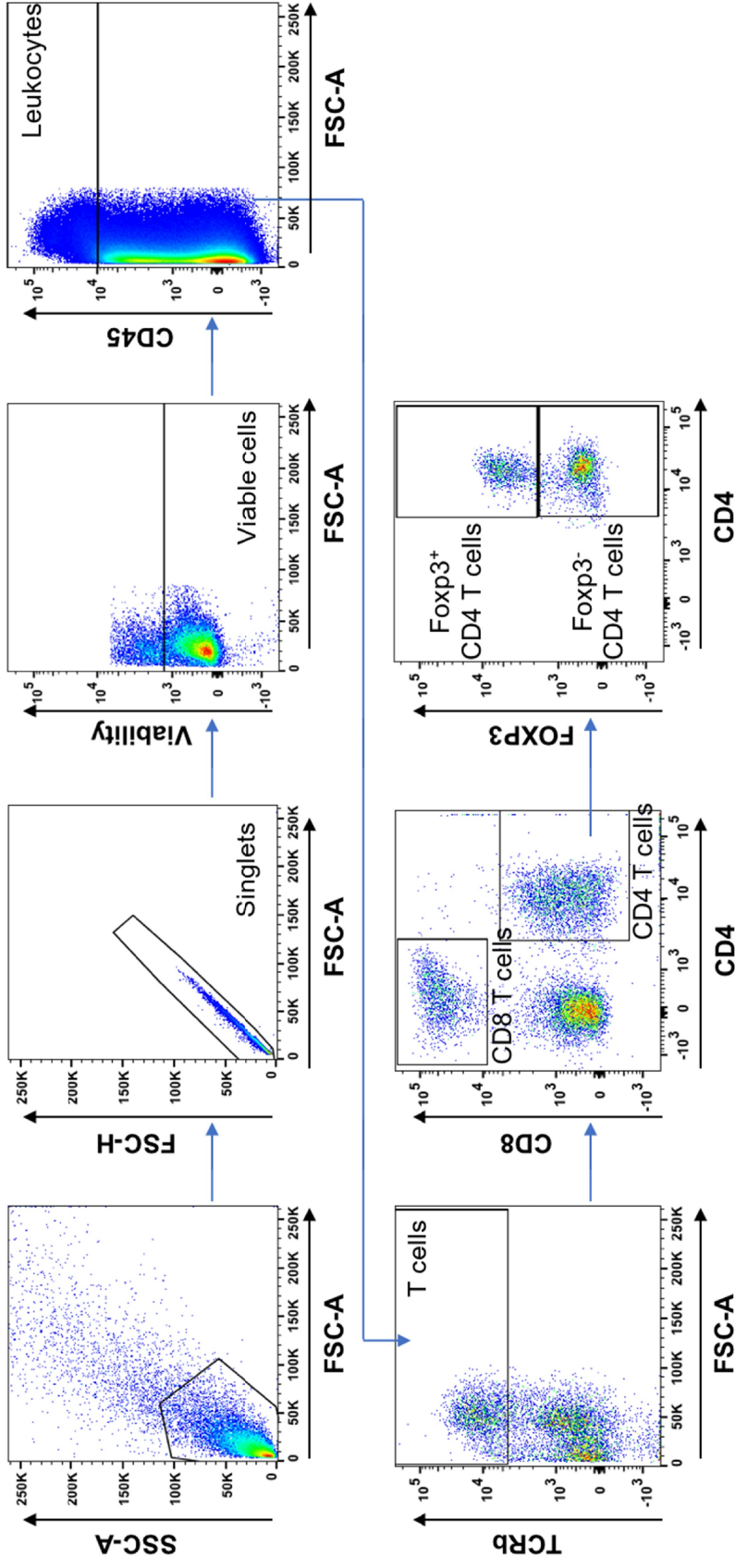

**B**

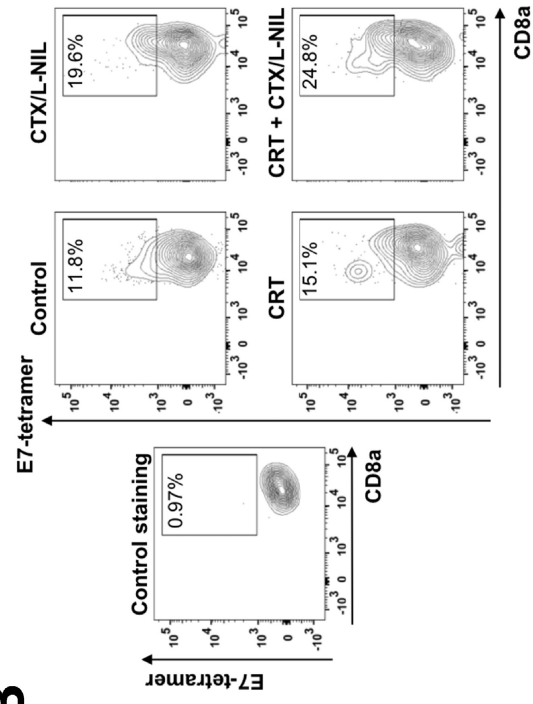

Supplement: Supplementary file 1 — Figure S1. The combination of CTX / L-NIL reverses the cold tumor microenvironment. Figure S2. CTX / L-NIL activates the immune microenvironment of CRT treated tumors. Figure S3. CTX/L-NIL improves CRT treatment effects in established HPV-negative tumors. Figure S4. Gating strategy for myeloid sub-types and inflammatory monocyte phenotyping. Figure S5. Systemic myeloid effects induced by CRT+CTX/L-NIL. Figure S6. Gating strategy for lymphocyte sub-types. Figure S7. Systemic lymphoid effects induced by CRT+CTX/L-NIL. Figure S8. CD8+ T cell phenotype in tumors. Table S1. Flow cytometry antibodies used for myeloid subset analysis. Table S2. Flow cytometry antibodies used for lymphocyte and CD8+ T cell subset analysis. Table S3. Immune pathway signature gene list used in gene expression analysis. Table S4. Immune cell type signature gene list used in gene expression analysis [68–70]. (ZIP 12061 kb) [file 40425_2018_485_MOESM1_ESM.zip › Fig. S6..pdf]

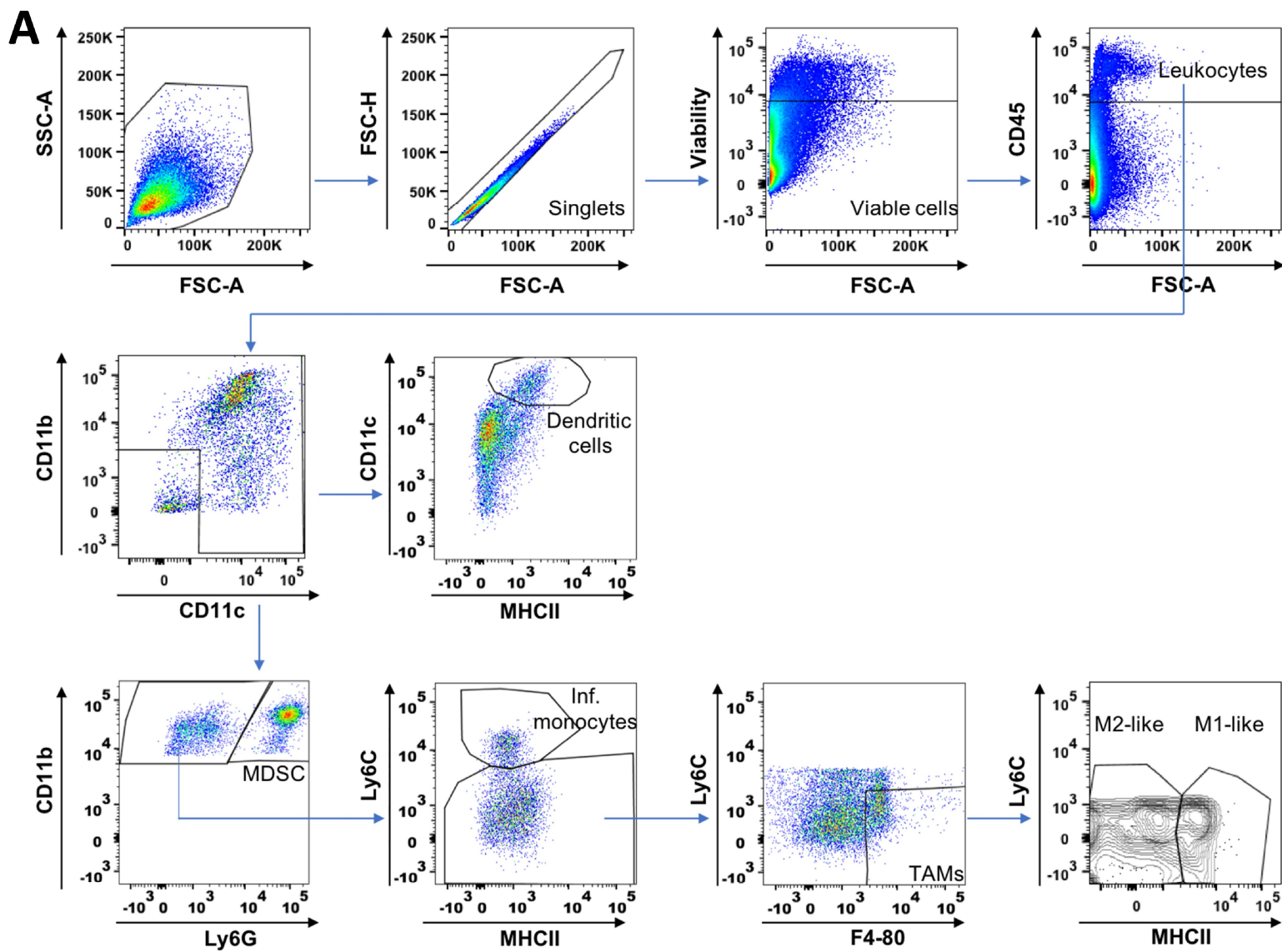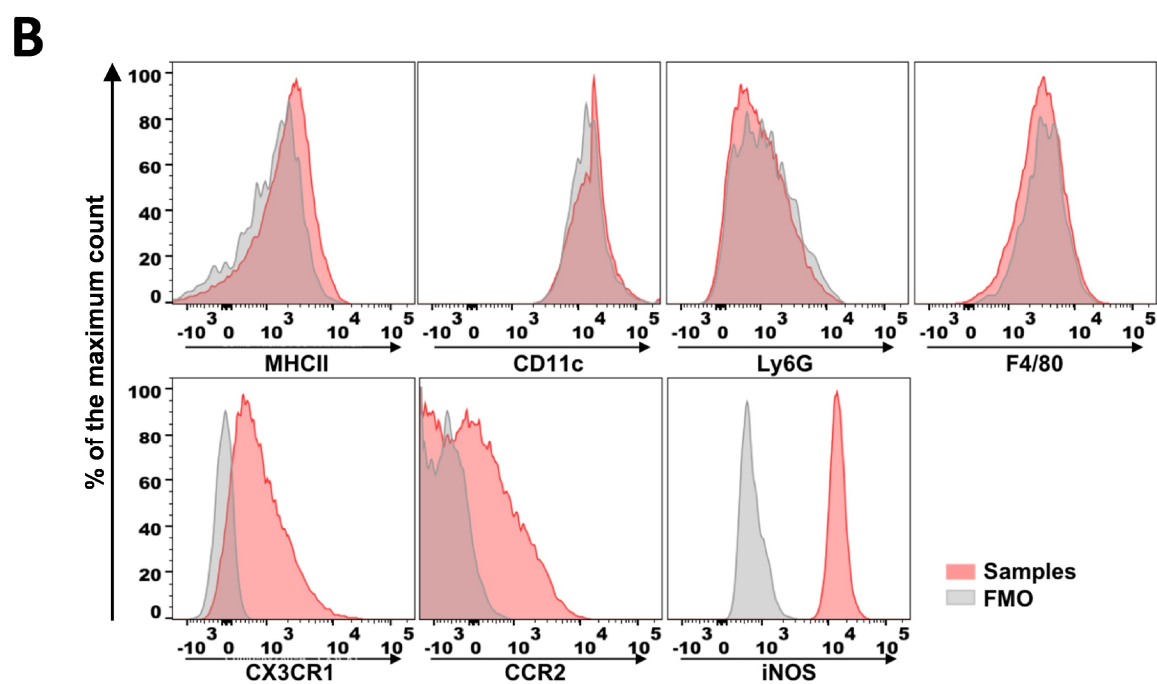

Supplement: Supplementary file 1 — Figure S1. The combination of CTX / L-NIL reverses the cold tumor microenvironment. Figure S2. CTX / L-NIL activates the immune microenvironment of CRT treated tumors. Figure S3. CTX/L-NIL improves CRT treatment effects in established HPV-negative tumors. Figure S4. Gating strategy for myeloid sub-types and inflammatory monocyte phenotyping. Figure S5. Systemic myeloid effects induced by CRT+CTX/L-NIL. Figure S6. Gating strategy for lymphocyte sub-types. Figure S7. Systemic lymphoid effects induced by CRT+CTX/L-NIL. Figure S8. CD8+ T cell phenotype in tumors. Table S1. Flow cytometry antibodies used for myeloid subset analysis. Table S2. Flow cytometry antibodies used for lymphocyte and CD8+ T cell subset analysis. Table S3. Immune pathway signature gene list used in gene expression analysis. Table S4. Immune cell type signature gene list used in gene expression analysis [68–70]. (ZIP 12061 kb) [file 40425_2018_485_MOESM1_ESM.zip › Fig. S4..pdf]

## dLNs

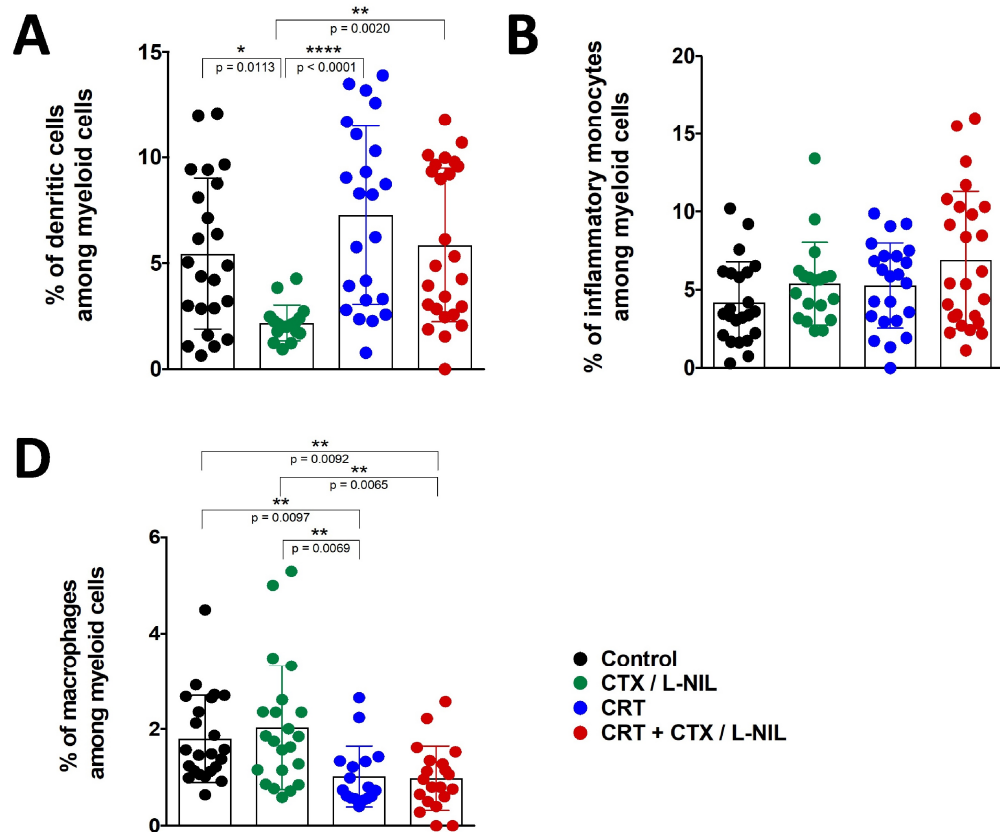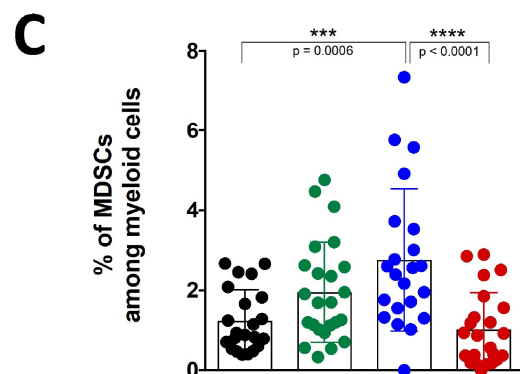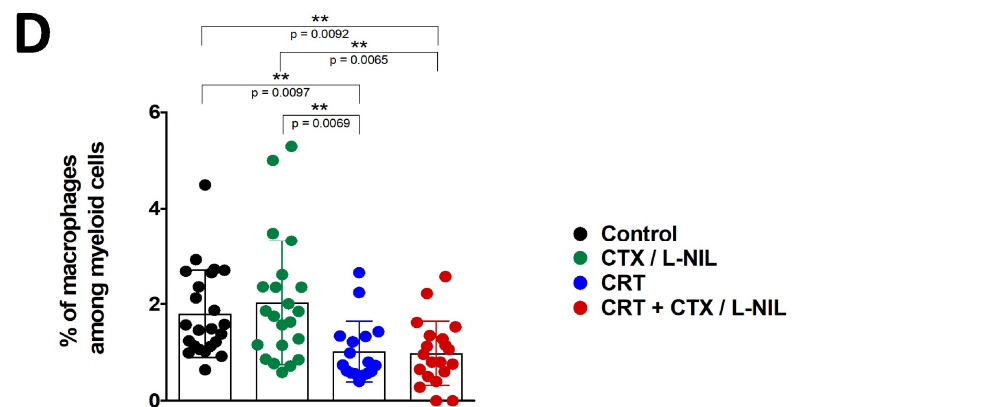

## Spleen

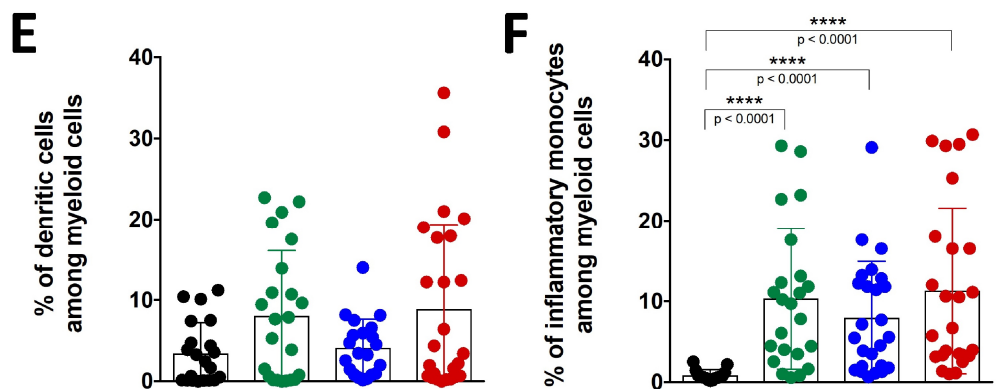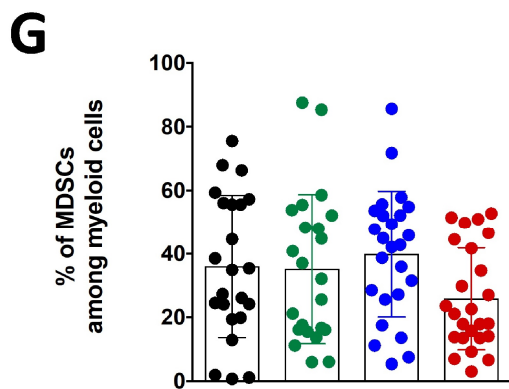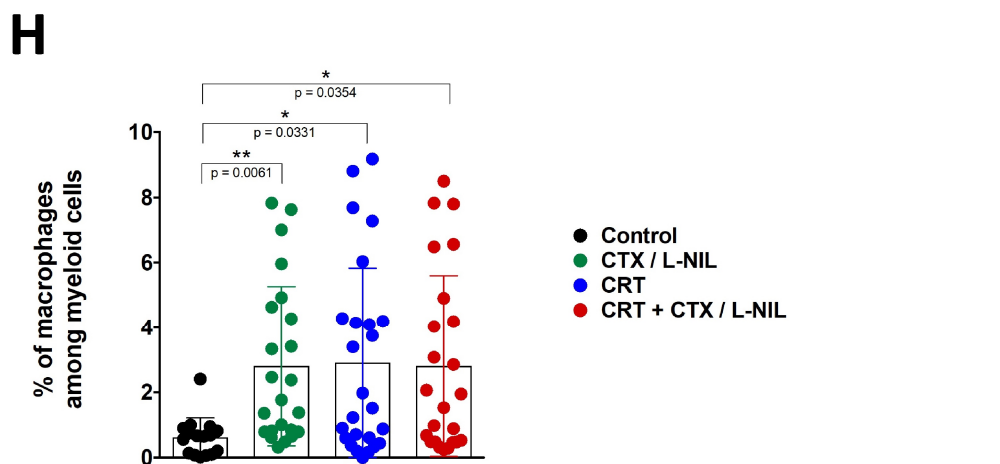

Supplement: Supplementary file 1 — Figure S1. The combination of CTX / L-NIL reverses the cold tumor microenvironment. Figure S2. CTX / L-NIL activates the immune microenvironment of CRT treated tumors. Figure S3. CTX/L-NIL improves CRT treatment effects in established HPV-negative tumors. Figure S4. Gating strategy for myeloid sub-types and inflammatory monocyte phenotyping. Figure S5. Systemic myeloid effects induced by CRT+CTX/L-NIL. Figure S6. Gating strategy for lymphocyte sub-types. Figure S7. Systemic lymphoid effects induced by CRT+CTX/L-NIL. Figure S8. CD8+ T cell phenotype in tumors. Table S1. Flow cytometry antibodies used for myeloid subset analysis. Table S2. Flow cytometry antibodies used for lymphocyte and CD8+ T cell subset analysis. Table S3. Immune pathway signature gene list used in gene expression analysis. Table S4. Immune cell type signature gene list used in gene expression analysis [68–70]. (ZIP 12061 kb) [file 40425_2018_485_MOESM1_ESM.zip › Fig. S5..pdf]

**A**

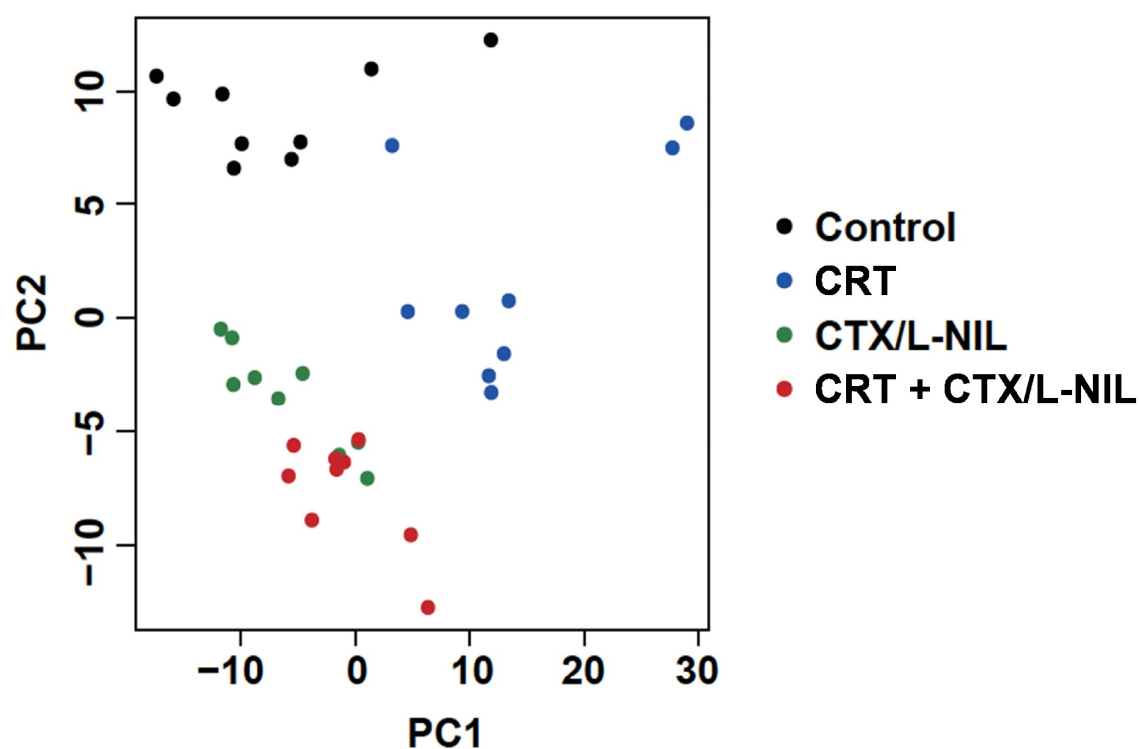

**B**

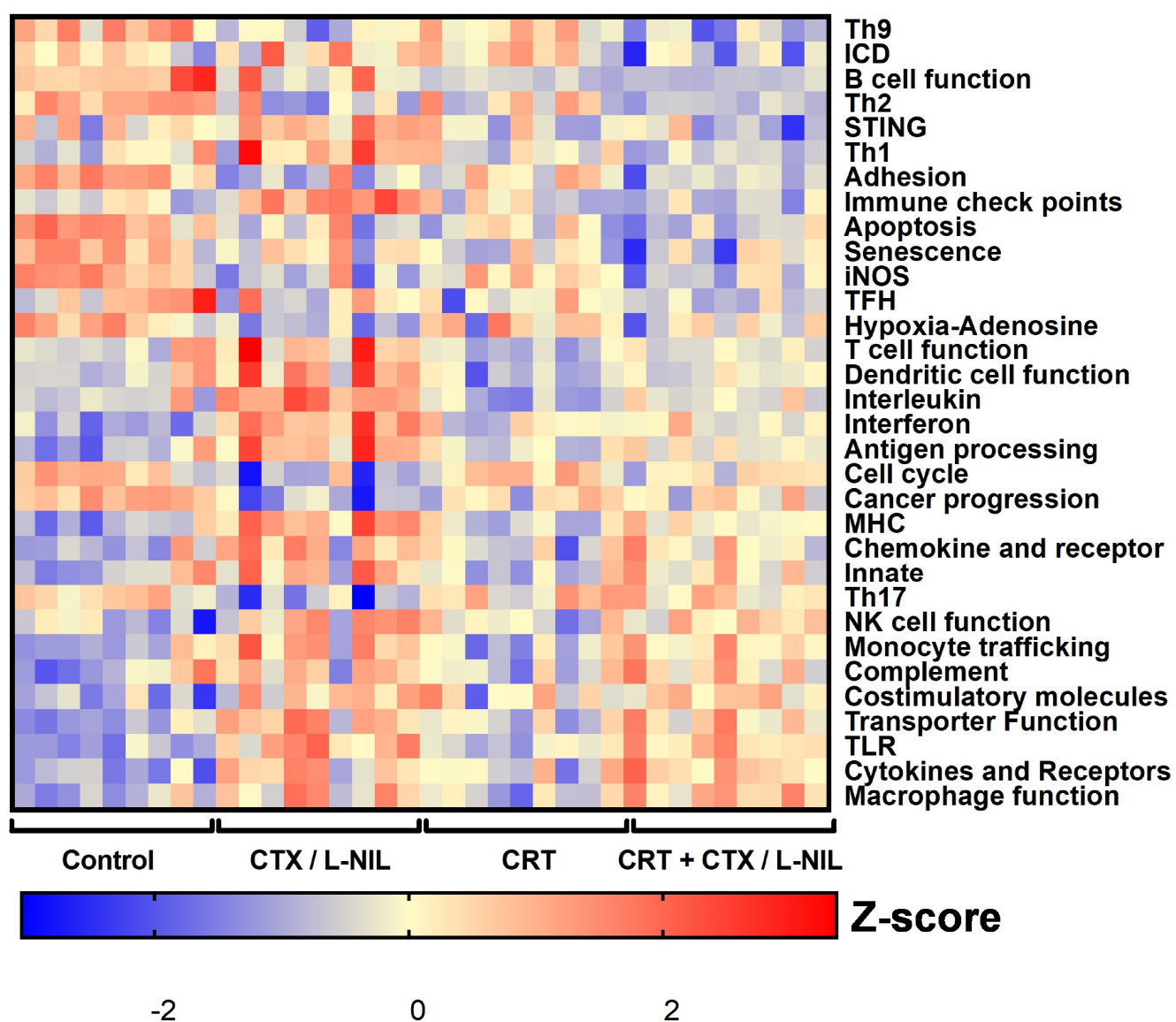

Supplement: Supplementary file 1 — Figure S1. The combination of CTX / L-NIL reverses the cold tumor microenvironment. Figure S2. CTX / L-NIL activates the immune microenvironment of CRT treated tumors. Figure S3. CTX/L-NIL improves CRT treatment effects in established HPV-negative tumors. Figure S4. Gating strategy for myeloid sub-types and inflammatory monocyte phenotyping. Figure S5. Systemic myeloid effects induced by CRT+CTX/L-NIL. Figure S6. Gating strategy for lymphocyte sub-types. Figure S7. Systemic lymphoid effects induced by CRT+CTX/L-NIL. Figure S8. CD8+ T cell phenotype in tumors. Table S1. Flow cytometry antibodies used for myeloid subset analysis. Table S2. Flow cytometry antibodies used for lymphocyte and CD8+ T cell subset analysis. Table S3. Immune pathway signature gene list used in gene expression analysis. Table S4. Immune cell type signature gene list used in gene expression analysis [68–70]. (ZIP 12061 kb) [file 40425_2018_485_MOESM1_ESM.zip › Fig. S2..pdf]

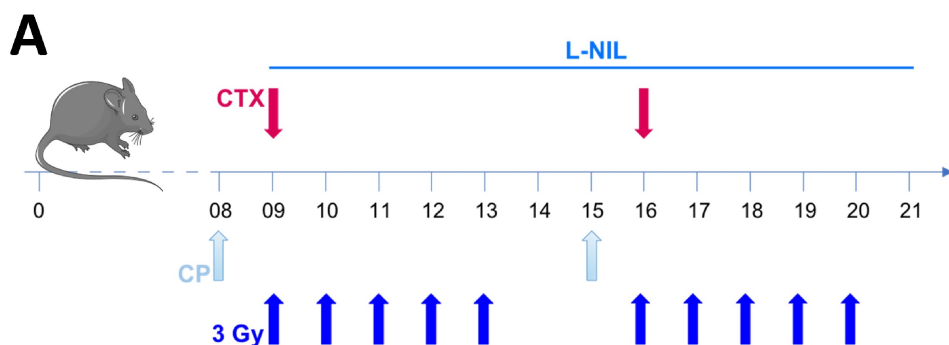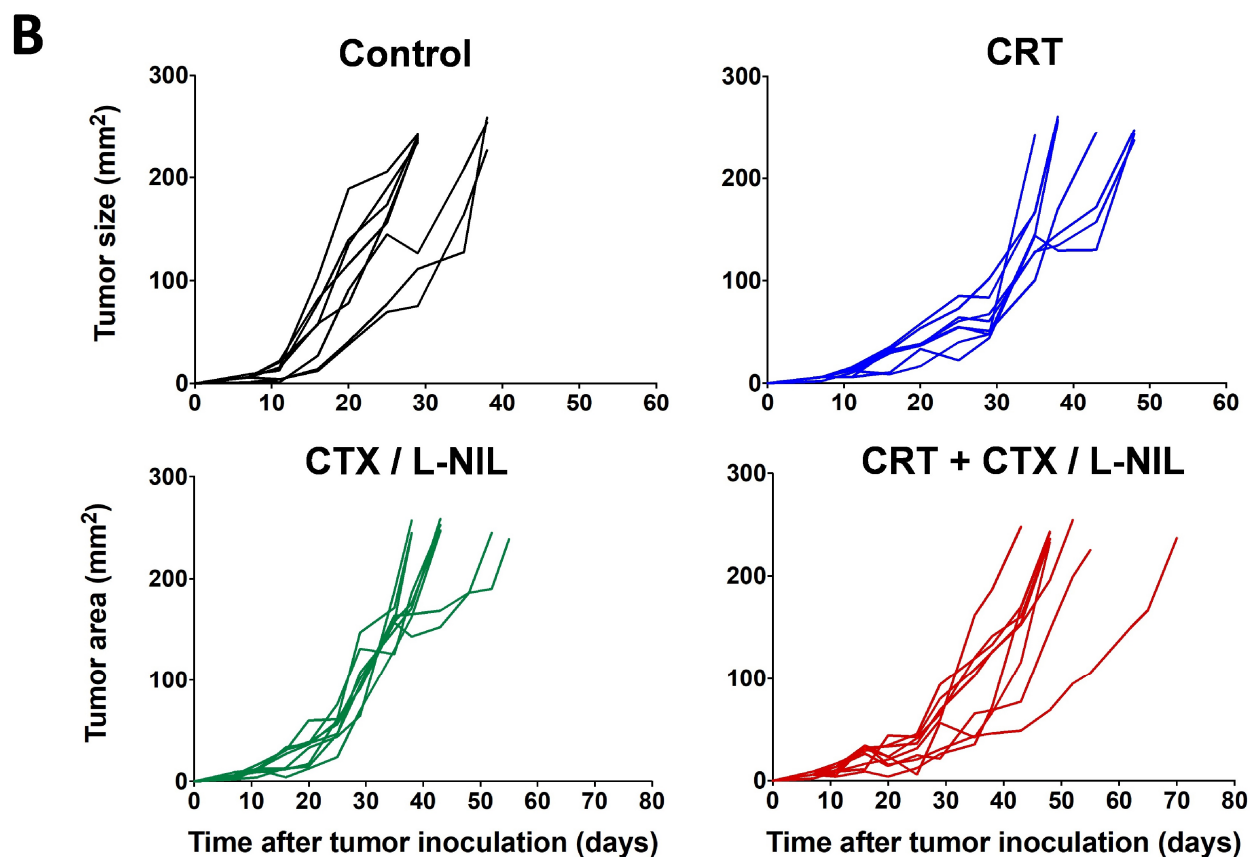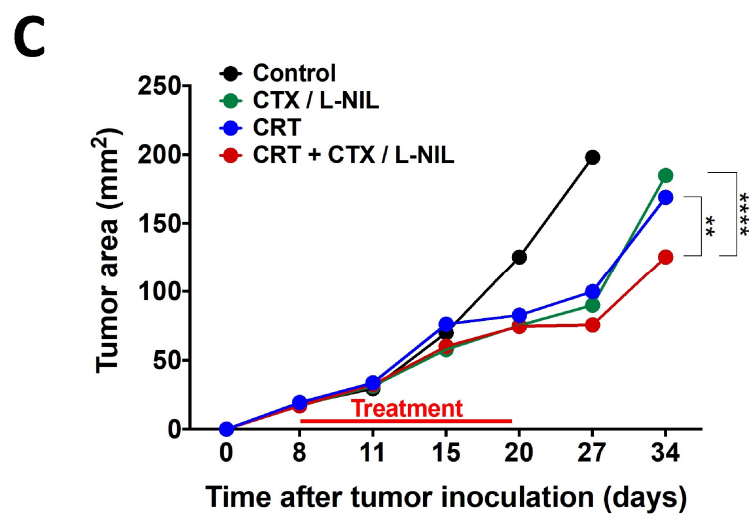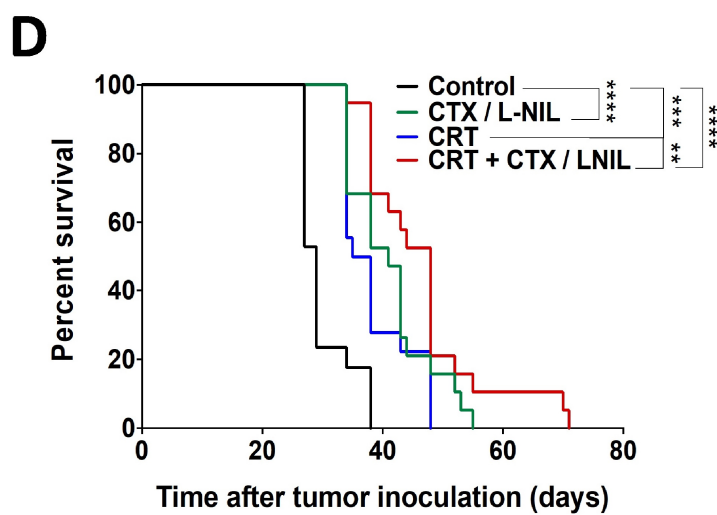

Supplement: Supplementary file 1 — Figure S1. The combination of CTX / L-NIL reverses the cold tumor microenvironment. Figure S2. CTX / L-NIL activates the immune microenvironment of CRT treated tumors. Figure S3. CTX/L-NIL improves CRT treatment effects in established HPV-negative tumors. Figure S4. Gating strategy for myeloid sub-types and inflammatory monocyte phenotyping. Figure S5. Systemic myeloid effects induced by CRT+CTX/L-NIL. Figure S6. Gating strategy for lymphocyte sub-types. Figure S7. Systemic lymphoid effects induced by CRT+CTX/L-NIL. Figure S8. CD8+ T cell phenotype in tumors. Table S1. Flow cytometry antibodies used for myeloid subset analysis. Table S2. Flow cytometry antibodies used for lymphocyte and CD8+ T cell subset analysis. Table S3. Immune pathway signature gene list used in gene expression analysis. Table S4. Immune cell type signature gene list used in gene expression analysis [68–70]. (ZIP 12061 kb) [file 40425_2018_485_MOESM1_ESM.zip › Fig. S3..pdf]

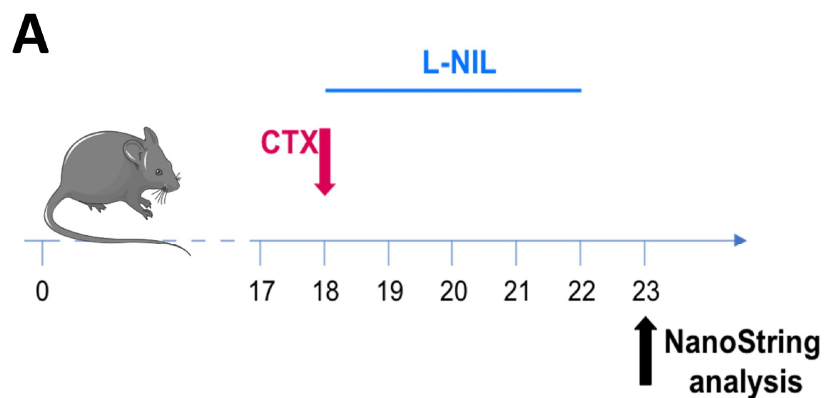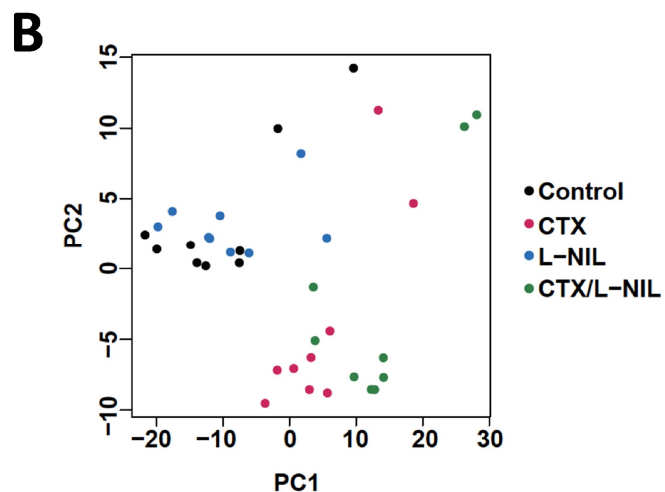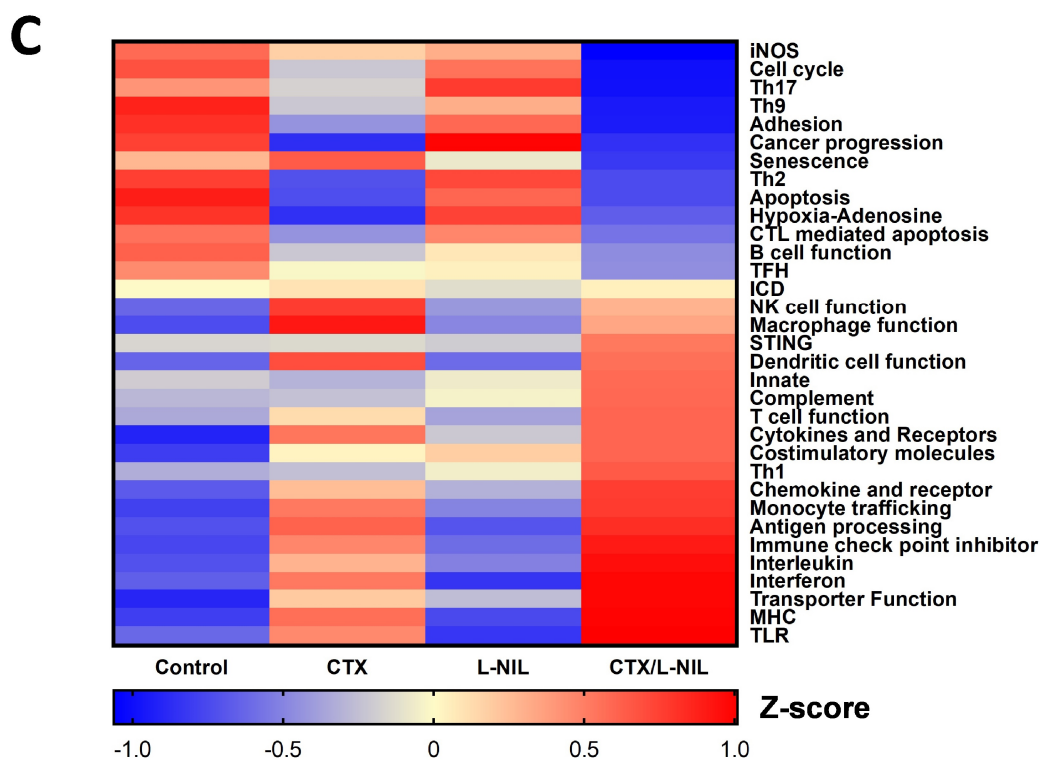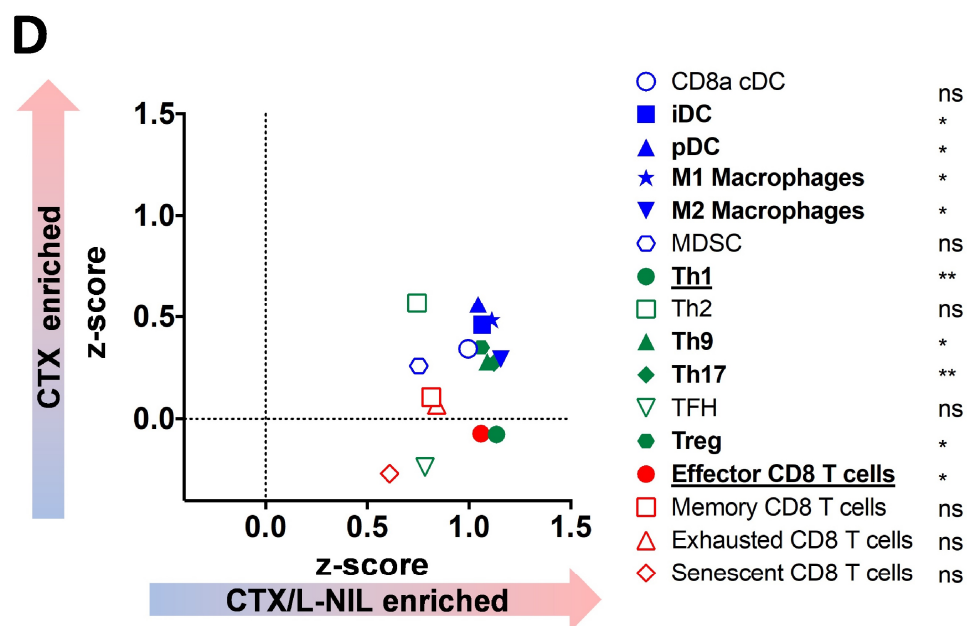

Supplement: Supplementary file 1 — Figure S1. The combination of CTX / L-NIL reverses the cold tumor microenvironment. Figure S2. CTX / L-NIL activates the immune microenvironment of CRT treated tumors. Figure S3. CTX/L-NIL improves CRT treatment effects in established HPV-negative tumors. Figure S4. Gating strategy for myeloid sub-types and inflammatory monocyte phenotyping. Figure S5. Systemic myeloid effects induced by CRT+CTX/L-NIL. Figure S6. Gating strategy for lymphocyte sub-types. Figure S7. Systemic lymphoid effects induced by CRT+CTX/L-NIL. Figure S8. CD8+ T cell phenotype in tumors. Table S1. Flow cytometry antibodies used for myeloid subset analysis. Table S2. Flow cytometry antibodies used for lymphocyte and CD8+ T cell subset analysis. Table S3. Immune pathway signature gene list used in gene expression analysis. Table S4. Immune cell type signature gene list used in gene expression analysis [68–70]. (ZIP 12061 kb) [file 40425_2018_485_MOESM1_ESM.zip › Fig. S1..pdf]
